# BAHD1 haploinsufficiency results in anxiety-like phenotypes in male mice

**DOI:** 10.1371/journal.pone.0232789

**Published:** 2020-05-14

**Authors:** Renaud Pourpre, Laurent Naudon, Hamid Meziane, Goran Lakisic, Luc Jouneau, Hugo Varet, Rachel Legendre, Olivia Wendling, Mohammed Selloum, Caroline Proux, Jean-Yves Coppée, Yann Herault, Hélène Bierne

**Affiliations:** 1 Université Paris-Saclay, INRAE, AgroParisTech, Micalis Institute, Jouy-en-Josas, France; 2 Micalis Institute, Université Paris-Saclay, CNRS, INRAE, AgroParisTech, Jouy-en-Josas, France; 3 Institut Clinique de la Souris-ICS, Université de Strasbourg, CNRS, INSERM, PHENOMIN, Illkirch, France; 4 Université Paris-Saclay, INRAE, Virologie et Immunologie Moléculaires, Jouy-en-Josas, France; 5 Institut Pasteur, Bioinformatics and Biostatistics Hub, C3BI, USR 3756 IP CNRS, Paris, France; 6 Institut Pasteur, Transcriptome and Epigenome Platform, Biomics Pole, Paris, France; 7 Université de Strasbourg, CNRS, INSERM, Institut de Génétique Biologie Moléculaire et Cellulaire (IGBMC), UMR7104, U1268, Illkirch, France; Technion Israel Institute of Technology, ISRAEL

## Abstract

BAHD1 is a heterochomatinization factor recently described as a component of a multiprotein complex associated with histone deacetylases HDAC1/2. The physiological and patho-physiological functions of BAHD1 are not yet well characterized. Here, we examined the consequences of BAHD1 deficiency in the brains of male mice. While *Bahd1* knockout mice had no detectable defects in brain anatomy, RNA sequencing profiling revealed about 2500 deregulated genes in *Bahd1*^-/-^ brains compared to *Bahd1*^+/+^ brains. A majority of these genes were involved in nervous system development and function, behavior, metabolism and immunity. Exploration of the Allen Brain Atlas and Dropviz databases, assessing gene expression in the brain, revealed that expression of the *Bahd1* gene was limited to a few territories and cell subtypes, particularly in the hippocampal formation, the isocortex and the olfactory regions. The effect of partial BAHD1 deficiency on behavior was then evaluated on *Bahd1* heterozygous male mice, which have no lethal or metabolic phenotypes. *Bahd1*^+/-^ mice showed anxiety-like behavior and reduced prepulse inhibition (PPI) of the startle response. Altogether, these results suggest that BAHD1 plays a role in chromatin-dependent gene regulation in a subset of brain cells and support recent evidence linking genetic alteration of BAHD1 to psychiatric disorders in a human patient.

## Introduction

Epifactors are nuclear proteins that play a role in epigenetic regulation of gene expression by acting on the structure of chromatin [1]. Epifactors are usually assembled into multiprotein complexes interacting with DNA-binding transcription factors in order to activate or repress transcription. The recruitment at specific genes and the biological function of these complexes depend on subunit composition and association with cell-type specific interaction partners. A subfamily of chromatin-repressive complexes is defined by the presence of the histone deacetylases HDAC1 and HDAC2, and central subunits serving as scaffolds, such as MTA, Sin3a, RCOR, MIDEAS and RERE, in the NURD, SIN3A, COREST, MIDAC (for a review [2]) and RERE complexes [3], respectively. During our research on an infectious disease, we serendipitously identified the Bromo Adjacent Homology Domain containing 1 (BAHD1) protein as a central component of a novel complex associated with HDAC1/2 [4–6]. BAHD1 forms a scaffold with MIER proteins structurally related to the MTA and RERE proteins. BAHD1 shares with MTA and RERE a Bromo-Adjacent Homology domain (BAH), which is known to promote binding to nucleosomes [7], whereas the MTA, RERE, and MIER proteins contain the ELM2 and SANT domains that recruit HDAC1/2 [8–10].

Co-immunoprecipitation and colocalization experiments [4], as well as tandem-affinity purification of BAHD1-associated proteins in human HEK293 cells [6], indicate that BAHD1-MIER-HDAC1/2 form a chromatin-repressive complex with the histone lysine methyltransferase (KMT) G9a, as well as HP1 and MBD1 (which are readers of methylated histone H3 and methylated DNA, respectively) and CDYL and KAP1. In mouse embryonic cells, BAHD1 also co-immunoprecipitates with MIER, HDAC, G9a and CDYL [11]. Other studies identified the KMTs SETDB1 [4] and SUV39H1 [12] as BAHD1 binding partners. Importantly, all these proteins play a key role in heterochromatin formation. In line with this, the overexpression of BAHD1 in human cells induces large-scale chromatin condensation [4] and changes in the DNA methylation landscape [13]. It is worth noting that the C-terminal BAH domain of BAHD1 (the origin of the name of the protein) is required for BAHD1 colocalization with H3K27me3 [4], which is a mark of facultative heterochromatin, and was recently shown to be a reader of H3K27me3 [14]. Likewise, the BAH domains of the SHL and EBS proteins in the plant *Arabidopsis thaliana* are H3K27me3 readers involved in chromatin-dependent repression [15].

The *BAHD1* gene (originally named *KIAA0945*) was identified by sequencing a cDNA library from a human adult brain [16]. Since then, relatively few studies have examined the function of the product of this gene, which is present only in vertebrates. We have demonstrated that BAHD1 is a chromatin regulator [4] that plays a role in the infection of epithelial cells by the bacterial pathogen *Listeria monocytogenes* and that modulates expression of interferon-stimulated genes (ISG) upon infection [5, 17]. *Listeria* bacteria have the ability to cross the epithelial barriers of the intestine, placenta and brain, and it was of interest to study the role of BAHD1 in these organs. In a previous work, we showed that the deletion of the *Bahd1* gene in mice leads to a defect in placental growth, which is associated with a low fetal weight and a high mortality at birth [6]. Surviving adult mutants grow similarly to wild-type littermates but have a lower weight, hypocholesterolemia and a reduction in adipose tissue. In addition, Zhu and collaborators have reported a role for BAHD1 in controlling inflammation in the gut [18]. The purpose of the present work was to determine whether BAHD1 plays a role in the brain in murine models.

## Materials and methods

### Ethics statement

Mice were bred and maintained in the animal facilities of the Institut Clinique de la Souris (ICS, Illkirch, France) or INRAE (Jouy-en-Josas, France), which are accredited by the French Ministry for Superior Education and Research and the French Ministry of Agriculture (agreement #A67-218-37 and B78-720, respectively), and in accordance with the Directive of the European Parliament: 2010/63/EU, revising/replacing Directive 86/609/EEC and with French Law (Decree n° 2013–118 01 and its supporting annexes entered into legislation 01 February 2013) relative with the protection of animals used in scientific experimentation. All animal experiments were approved by local ethical committees (ICS Com’Eth S and INRAE COMETHEA, registered under the reference “C2EA– 35” and DAP14_06, respectively) and supervised in compliance with the European Community guidelines for laboratory animal care and use.

### Mouse lines, brain collection and tissue preparation

The heterozygous *Bahd1*-Het1 mice and homozygous *Bahd1-*KO mice (obtained by crossing *Bahd1*-Het1) have been described previously [5, 6]. The generation of the *Bahd1*-Het2 mouse line (C57BL/6N *Bahd1*^tm1B(KOMP)Wts1^) is described in the Supplementary information. *Bahd1-*KO and *Bahd1*-Het2 mice were bred and maintained in the animal facilities of the Institut Clinique de la Souris (ICS, Illkirch, France) and *Bahd1*-Het1 in the animal facilities of INRAE (Jouy-en-Josas, France) under pathogen-free conditions with free access to food and water. Throughout experiments mice were housed in the same climate-controlled stable with a 12h/12h dark-light cycle and handled identically. All the behavioral tests were performed between 9:00 AM and 4:00 PM. For wild type and *Bahd1*^*-/-*^ production, *Bahd1*^+/-^ mice were mated and the day on which a vaginal plug was found was designated 0.5. Genotyping was performed on genomic DNA isolated from tail biopsies with specific PCR, as described previously [5]. Full deficiency or haplodeficiency of *Bahd1* expression was verified by RT-qPCR. For collection of embryonic brains at E16.5, pregnant mice were killed by cervical dislocation and fetuses were delivered by caesarian section and transferred in phosphate buffer saline (PBS). They were killed by decapitation and tail biopsies were taken for genotyping by PCR. The brains were dissected and placed in RNAlater^™^ Stabilization Solution (Ambion AM7024) at 4°C, freezed in liquid nitrogen and stored at -80°C until RNA extraction for transcriptome experiments. For collection of adult *Bahd1*-KO brains, mice were killed by cervical dislocation, brains were dissected and cut sagittaly. Halves of the brains were immediately immerged in RNAlater^™^ and frozen at -80°C until they were submitted to RNA extraction transcriptome experiments. The other halves were fixed in 10% formalin and embedded in paraffin for histology, with three series of ten slides prepared from 5μm-thick paraffin sections performed on each brain. One slide of each series was stained with the haematoxylin and eosin staining (3 slides / brain), luxol fast blue and cresyl violet or Periodic-Acid-Schiff (PAS). Stained sections were digitalized using a slide scanner (Nanozoomer 2.0-HT, *Hamamatsu*, Japan). The seven other slides of each series were stored for eventual immunohistochemical staining.

### Behavioral tests

All the mice used in the study were males. We generated two independent cohorts, cohort 1 (WT *n* = 7; *Bahd1-*Het1 *n* = 7) and cohort 2 (WT *n* = 16; *Bahd1-*Het2 *n =* 9). A detailed description of tests to which each cohort was submitted, and statistics, are available in the Supplementary information.

### RNA-seq data and gene functional analysis

Total RNA was extracted from one hemisphere of adult mice brains (n = 3 per genotype) or from the whole brain of E16.5 embryos (n = 3 per genotype) using RNeasy Kit (Qiagen), according to the manufacturer’s instructions. The RNA Integrity Number median was 9.3 (range from 8.1 to 10; Bioanalyser Agilent). We purify polyadenylated mRNAs and build an RNA library, using TruSeq Stranded mRNA Sample Prep Kit (Illumina, #RS-122-9004DOC) as recommended by the manufacturer. Directional library were checked for concentration and quality on DNA chips first with the Bioanalyser Agilent, then with sensitive fluorescent-based quantitation assays ("Quant-It" assays kit and QuBit fluorometer, Invitrogen). The 12 cDNA libraries were sequenced using a HiSeq 2500 sequencer (Illumina) in 65 bases V4 single-end mode in three technical replicates. The procedures for Read mapping on the reference genome (from Ensembl-92 GRCm38 assembly), gene counting, normalization and statistical analyzes are available in the Supplementary information. Functional gene analysis was performed on protein-encoding genes with Ingenuity Pathway Analysis (Ingenuity® Systems, www.ingenuity.com, version 2018) software and DAVID (http://david.abcc.ncifcrf.gov, 2018 version 6.8 [19]). The range of *p*-values were calculated using the right-tailed Fisher's Exact Test, which compares the number of user-specified genes to the total number of occurrences of these genes in the respective functional/pathway annotations stored in the Ingenuity Pathways Knowledge or DAVID databases. Gene clusters with less than 10 genes were not considered in the analysis.

### Quantification of transcript levels by real-time quantitative PCR (RT-QPCR)

We used RNA extracted from half brains of *Bahd1*-KO and -WT mice (n = 4 per genotype) or from the whole brain of 7 *Bahd1*-Het1 mice and WT littermates (n = 7 per genotype). Genomic DNA was removed by treatment with TURBO DNA-free TM kit (Ambion). cDNAs were generated from 1 μg total RNA using the AffinityScript QPCR cDNA Synthesis Kit (Agilent). Quantitative Real-Time PCR was performed on StepOne Plus Real-Time PCR Systems (Applied Biosystems) using KAPA SYBR® FAST qPCR Master Mix (2X) Kit (Kapa Biosystems), as specified by the supplier. Each reaction was performed in triplicate. Data were analyzed by the ΔΔCt method. Target gene expression data were normalized to the relative expression of mouse *Ywhaz* gene. *Hrpt* gene was used as a control gene. Statistical significance of the difference in mean expression of genes was evaluated using the Student *t*-test; *p*<0.05 was considered significant. Primer sets are provided in the Supplementary information.

### Databases and gene expression analysis tools

The databases used in our study were the NCBI database (Genbank, UniGene), version 18 of human protein atlas (https://www.proteinatlas.org/) [20] and versions 2019 of mouse encode database [21], Allen Brain Atlas (ABA) for mouse brain (https://mouse.brain-map.org/) [22] and human brain (http://www.brain-map.org/) [23], DropViz (http://dropviz.org/) [24] and Harmonizome (https://amp.pharm.mssm.edu/Harmonizome/) [25] databases. In regards to ABA ISH dataset [22], anatomically comprehensive expression patterns of over 16,000 genes were obtained at cellular resolution in the 56-day-old male C57BL/6J mouse brain. ISH photomicrographs of the *Bahd1* probe (RP_050421_03_A07) were manually analyzed for the expression of *Bahd1* in brain areas, and inspected at multiple magnification levels and evaluated for gene expression consistency. *Bahd1* expression density and level were scored with reference to the ABA expression heat mask in 12 anatomical subdivisions: isocortex olfactory areas, hippocampal formation, cortical subplate, striatum, pallidum, thalamus, hypothalamus, midbrain, pons, medulla, cerebellum. The relative expression of a gene is given as a quantity of energy expression in each brain structure.

## Results

### Histological study of the brain in *Bahd1* knockout mice

*Bahd1* knockout (KO) mice and wild type (WT) mice described in our previous study [6] were euthanized at approximately 17 months of age (n = 4 per genotype). Their brains were sagittally cut, with one half being immediately and rapidly frozen at -80°C for subsequent transcriptomic analysis, while the other half was fixed in formalin and embedded in paraffin for histopathological analysis. To evaluate the potential pathological changes in the brains of *Bahd1*-KO mutants, three histological stains were used: haematoxylin and eosin, for classic staining of the tissue structure; Periodic acid-Schiff (PAS) to label the glycoproteins; and Luxol fast blue and cresyl violet, to observe possible neuronal lesions (loss of Nissl substance) or axon degeneration (myelin degradation). None of these stains showed any morphological differences or aberrant cell structures in different brain sections from homozygous mutants compared with those from WT mouse brains. Representative images of these stains in different regions of the brains of each genotype are shown in S1 Fig. Overall, no histopathological abnormality was detected in the *Bahd1-*KO mutant brains upon comparison with WT brains.

### Changes in the cerebral transcriptional landscape in *Bahd1* knockout adult mice

**Fig 1:**
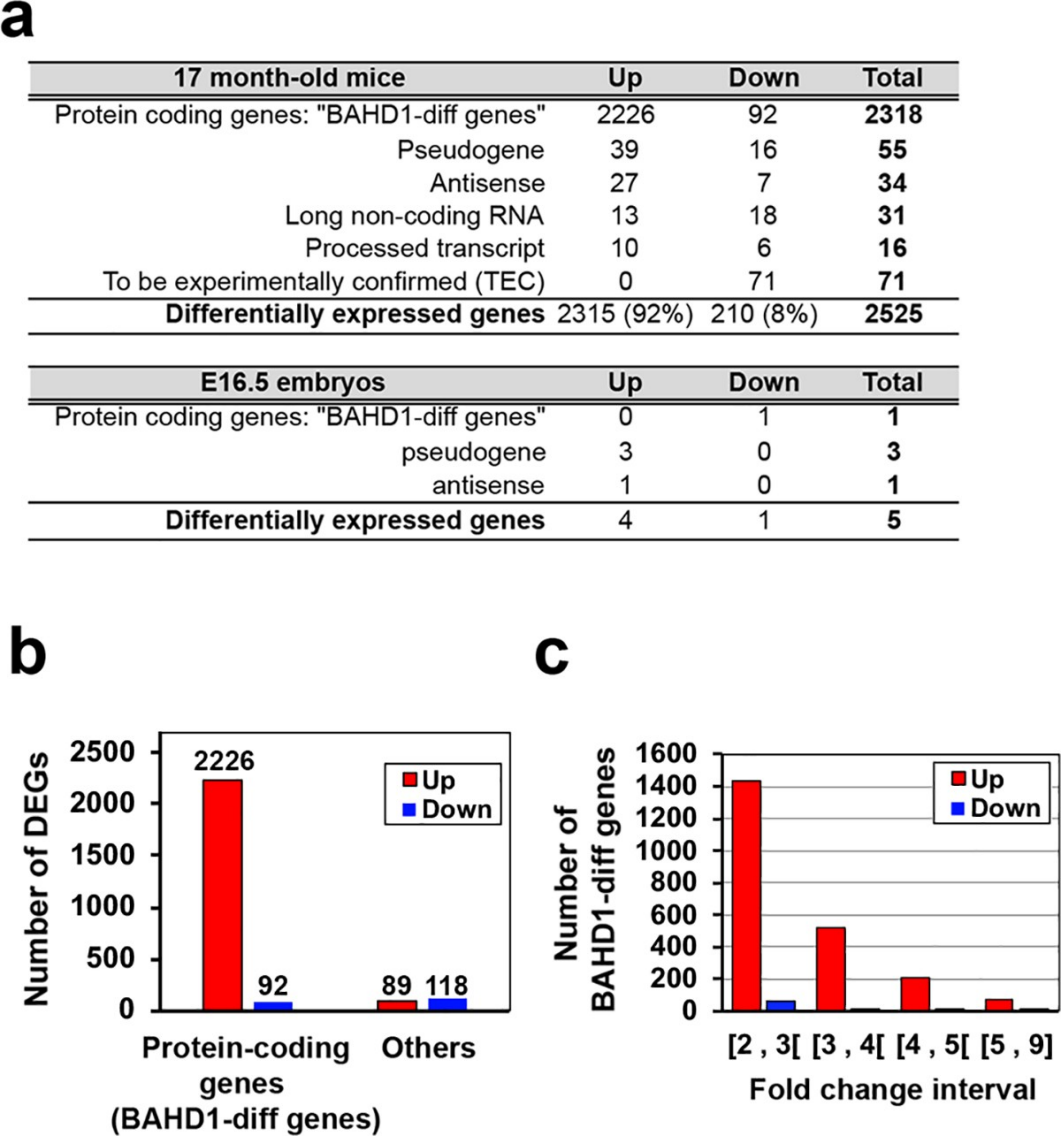
BAHD1 deficiency reshapes gene expression in the adult murine brain. (**a**) Summary of the transcriptomic analysis of *Bahd1*^-/-^ and *Bahd1*^+/+^ brains from 17-month-old mice or embryos (E16.5). DEGs are categorized into gene types. (**b**) Number of upregulated (fold change ≥2) or downregulated (or ≤-2) genes. The number of protein-coding genes (termed “BAHD1-diff genes”) and that of other gene categories (“Others”) are indicated. (**c**) Number of BAHD1-diff genes per interval of fold change.

To investigate whether BAHD1 could play a role in transcriptional regulation in the brain, we performed RNA-seq analyses on the other half of brains examined in histology (three of each genotype, *Bahd1*-WT *vs*. *Bahd1*-KO). Hierarchical clustering and principal component analysis (PCA) revealed that the absence of *Bahd1* changed the gene expression profile in the KO mice compared to that in the WT brains (S2A Fig). The *Bahd1*-null mutation modified the expression of 2525 transcripts (Benjamini-Hochberg adjusted *p*-value <0.05) with a change in expression greater or less than two-fold (Fig 1A and S1 Table). Most genes (92%) were overexpressed in the *Bahd1*-KO brains compared to that in the WT brains, which is consistent with the role played by BAHD1 in the formation of repressive chromatin. We also wished to determine whether the deregulation of BAHD1 could modify the expression of brain genes during embryonic development. To address this point, we analyzed the transcriptomes of *Bahd1*-WT and -KO embryonic brains (n = 3 per genotype) at embryonic day 16.5 (E16.5). The results of this analysis showed that the samples were not grouped separately according to the genotype (S2B Fig), and in addition to *Bahd1*, only the expression of three pseudogenes and one antisense gene were significantly changed (Benjamini-Hochberg adjusted *p*-value <0.05, threshold ≥2 or ≤-2-fold) (Fig 1A). Because one of the embryos was possibly an outlier, the analysis was redone in the absence of this sample (S2C Fig). Overall, this removal did not notably change the result, as only two protein-encoding genes and one pseudogene were additionally deregulated. These results showed that complete BAHD1 deficiency remodels the brain transcriptome in adult mice but does not significantly affect gene expression in the embryonic brain at a late fetal stage (E16.5).

**Fig 2:**
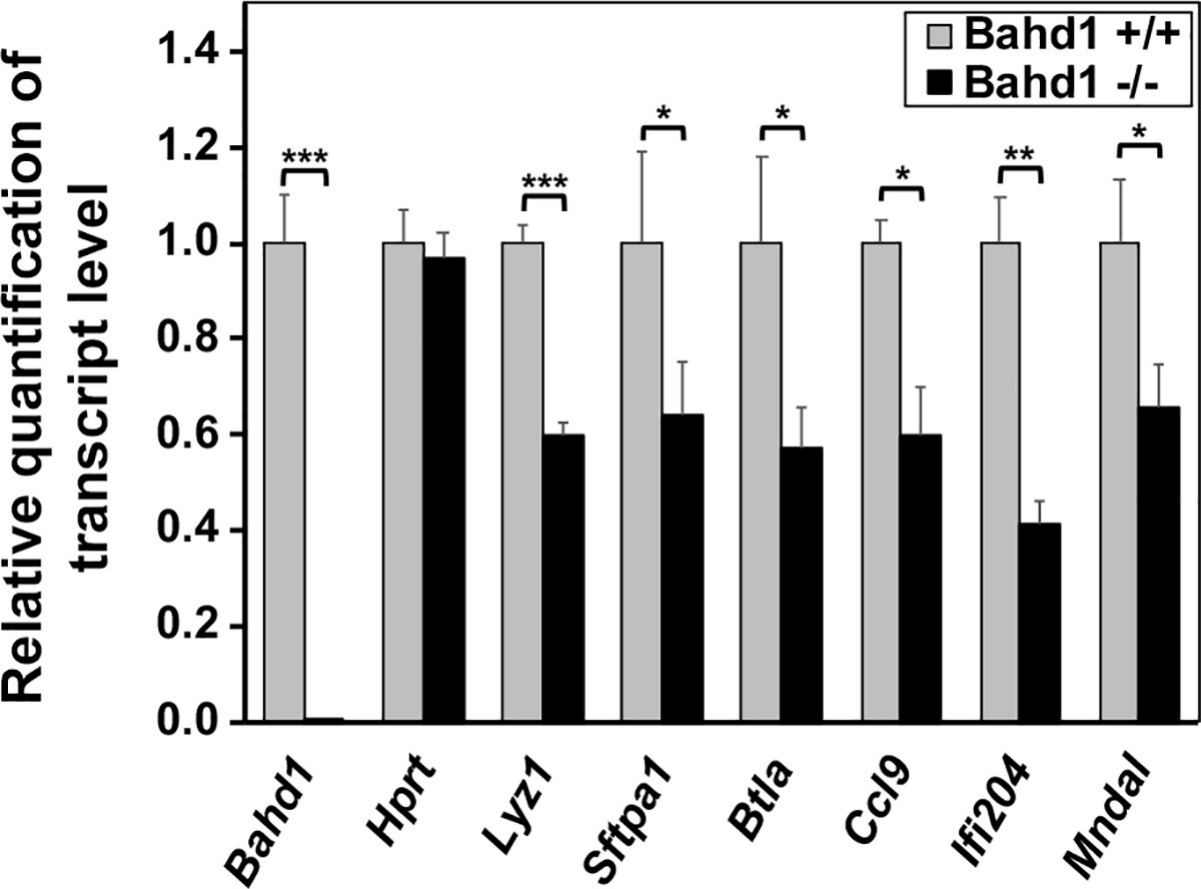
A set of immunity-related genes is downregulated in *Bahd1*^−/−^ brains in comparison with *Bahd1*^+/+^ brains. Relative transcript levels of immunity-related genes in *Bahd1*^−/−^ brains relative to that in *Bahd1*^+/+^ brains (n = 3 for each genotype), as determined by RT-qPCR (mean ± S.E.M.; * p<0.05, ** p<0.01, *** p<0.001).

The majority of the differentially-expressed genes (DEGs) in adult brains encoded proteins (2226 upregulated and 92 downregulated protein-coding genes), hereafter referred to as the “BAHD1-diff genes” (Fig 1B). It should be noted that the number of BAHD1-diff genes was large, but the variation in the expression of each gene was low. In fact, the vast majority of genes (~ 70%) were deregulated between only 2- and 3-fold (Fig 1C). To investigate the functional significance of “BAHD1-diff genes”, we used the Ingenuity Pathway Analysis (IPA) software to group the genes into categories according to “Biological Functions and Diseases”. Upregulated genes grouped into 75 highly significant categories (selected according to *p*<0.0005 and at least 10 genes per category, S2 Table), of which 22 (29%) were involved in the development, growth or death of brain cells, functions of the nervous system (e.g., behavior, cognition, or mood disorders) and/or the amount of neurotransmitters (Table 1A). In addition, three gene clusters were associated with metabolism: the concentration of lipids, obesity and the oxidation of carbohydrates. Downregulated genes grouped into 16 significant categories (S3 Table), of which 10 (62%) encompassed genes involved in bacterial infections, immunity and/or inflammation (Table 1B). IPA results combined with a functional annotation with the DAVID software [19] and a bibliography search showed that half of the downregulated genes were involved in infection, immunity and/or host defense (S4 Table). Of note, 26 of these genes were identified as interferon-response genes in the interferome database [26] (Table 2). Real-time quantitative PCR (RT-qPCR) analysis validated the decreased expression of representative genes encoding important mediators of innate immunity, including lysozyme 1 (*Lyz1*), B and T lymphocyte attenuator (*Btla*), surfactant-associated protein A1 (*Sftpa1*), chemokine (C-C motif) ligand 9 (*Ccl9*), interferon-activable protein 204 (*Ifi204*), and myeloid nuclear differentiation antigen-like (*Mndal*). The results were consistent with the results of the RNA-seq analysis (Fig 2). To conclude, at the level of global brain-wide analyses, the dominant feature of the transcriptional variation between the brains of *Bahd1*-KO and *Bahd1*-WT mice was the overexpression of genes involved in neurological functions, behavior, cognition and learning, along with the under-expression of genes involved in innate immunity and bacterial diseases.

**Table 1:** Subset of biological functions and diseases associated with BAHD1-diff genes.

**a. Diseases and Functions Annotation (Up-regulated genes)** ^**a**^	**# genes**	***p*-Value**
**Nervous system development and functions; Neurological diseases**		
Growth of neurites	84	1.83E-06
Outgrowth of neurons	71	2.18E-06
Outgrowth of neurites	70	3.01E-06
Proliferation of neuronal cells	89	6.41E-06
Cell death of cerebral cortex cells	46	3.53E-05
Behavior	106	4.18E-05
Neuritogenesis	79	5.29E-05
Cognition	54	6.58E-05
Quantity of monoamines	31	7.33E-05
Cell death of brain	54	1.09E-04
Development of neurons	97	1.11E-04
Cell death of brain cells	51	1.20E-04
Release of catecholamine	20	1.31E-04
Learning	49	1.48E-04
Cell death of central nervous system cells	54	1.50E-04
Quantity of neurotransmitters	26	1.94E-04
Motor function	26	2.88E-04
Cell death of neuroblastoma cell lines	41	3.03E-04
Quantity of catecholamine	23	3.09E-04
Mood Disorders	75	4.61E-04
Seizures	63	4.67E-04
Cell death of cortical neurons	34	4.71E-04
**Lipid and carbohydrate metabolism; Metabolic diseases**	** **	** **
Concentration of lipid	123	5.73E-05
Obesity	89	4.15E-04
Oxidation of carbohydrate	14	2.48E-04
**b. Diseases or Functions Annotation (Down-regulated genes)**	**# genes**	***p*-Value**
**Infectious diseases, immunity and inflammation**	** **	** **
Bacterial Infections	11	1.10E-07
Systemic autoimmune syndrome	19	2.98E-07
Cell movement of myeloid cells	12	1.04E-06
Leukocyte migration	15	1.85E-06
Cell movement of phagocytes	11	6.28E-06
Inflammation of organ	18	9.51E-06
Inflammation of absolute anatomical region	15	2.17E-05
Inflammation of body cavity	13	3.01E-05
Psoriasis	11	3.56E-05
Cell movement of leukocytes	12	6.23E-05

^**a**^ IPA categories with ≥10 genes; *p*<0.0005. All categories are described in S2 and S3 Tables.

**Table 2:** Interferon-response genes downregulated in *Bahd1* knockout brains.

Gene name	Fold change	adj *p* value	Gene description
*Atp6v0d2*	-3.27	2.2E-02	ATPase, H+ transporting, lysosomal V0 subunit D2
*Ccl9*	-2.04	1.3E-02	chemokine (C-C motif) ligand 9
*Ccr1*	-2.54	4.9E-03	chemokine (C-C motif) receptor 1
*Cd36*	-2.70	1.1E-02	CD36 antigen
*Fmo3*	-4.94	3.8E-03	flavin containing monooxygenase 3
*Ifgga4*	-3.35	1.1E-02	Interferon-gamma-inducible GTPase Ifgga4 protein
*Ifi203*	-2.25	1.4E-03	interferon activated gene 203
*Ifi204*	-2.79	9.9E-04	interferon activated gene 204
*Ifi213*	-2.80	1.4E-02	interferon activated gene 213
*Ifi44*	-2.08	7.3E-03	interferon-induced protein 44
*Ifitm7*	-2.11	4.6E-02	interferon induced transmembrane protein 7
*Igsf6*	-2.04	7.3E-03	immunoglobulin superfamily, member 6
*Iigp1*	-2.15	9.6E-03	interferon inducible GTPase 1
*Il18r1*	-3.39	1.9E-02	interleukin 18 receptor 1
*Lyz1*	-4.83	4.7E-03	lysozyme 1
*Mid1*	-3.50	2.7E-08	E3 ubiquitin-protein ligase Midline-1
*Mki67*	-2.89	3.1E-03	proliferation marker protein Ki-67
*Mmp8*	-2.72	7.7E-03	matrix metallopeptidase 8
*Mndal*	-2.46	1.2E-04	Myeloid cell nuclear differentiation antigen-like protein
*S100a8*	-2.21	1.8E-02	S100 calcium binding protein A8 (calgranulin A)
*Sell*	-2.12	3.5E-02	selectin, lymphocyte
*Serpinb2*	-3.04	3.9E-02	serine (or cysteine) peptidase inhibitor, clade B, member 2
*Tinag*	-3.64	2.3E-02	tubulointerstitial nephritis antigen
*Tlr1*	-2.27	9.9E-03	toll-like receptor 1
*Top2a*	-2.13	4.3E-02	topoisomerase (DNA) II alpha
*Trim30a*	-2.10	1.9E-03	tripartite motif-containing 30A

### Expression profiling of the BAHD1 encoding gene in the mouse and human brain

The study of the entire transcriptome of the brain only detected global transcriptional changes resulting from direct and indirect effects of the *Bahd1*-null mutation, within a very complex organ composed of billions of diverse cells with highly specialized functions. Therefore, a prerequisite for targeted local analyses of BAHD1 function in the brain was to obtain a better knowledge of the expression of the gene coding for BAHD1. To this end, we surveyed publicly available transcriptomic datasets and *in situ* hybridization (ISH) datasets. Transcriptome databases derived from human [20] and mouse [21] tissue samples indicated that the BAHD1-encoding gene is expressed at low levels in a wide range of tissues in both human and mouse and does not belong to a class of genes that are specifically enriched in the brain, such as *Grin1* (encoding the NMDA-type subunit 1 of the ionotropic glutamate receptor) (S3 and S4 Figs). By comparison, the low-level ubiquitous expression of *Bahd1* is comparable to that of the gene encoding RCOR1, a scaffold protein of the HDAC1/2-associated COREST complex, which is known for its role in the epigenetic programming of neuronal cells [27]. However, while the level of the expression of *Rcor1* was decreased in the central nervous system (CNS) of adult mice compared to that in embryonic mice, the expression of *Bahd1* does not appear to vary according to the stage of development (S4 Fig).

**Fig 3:**
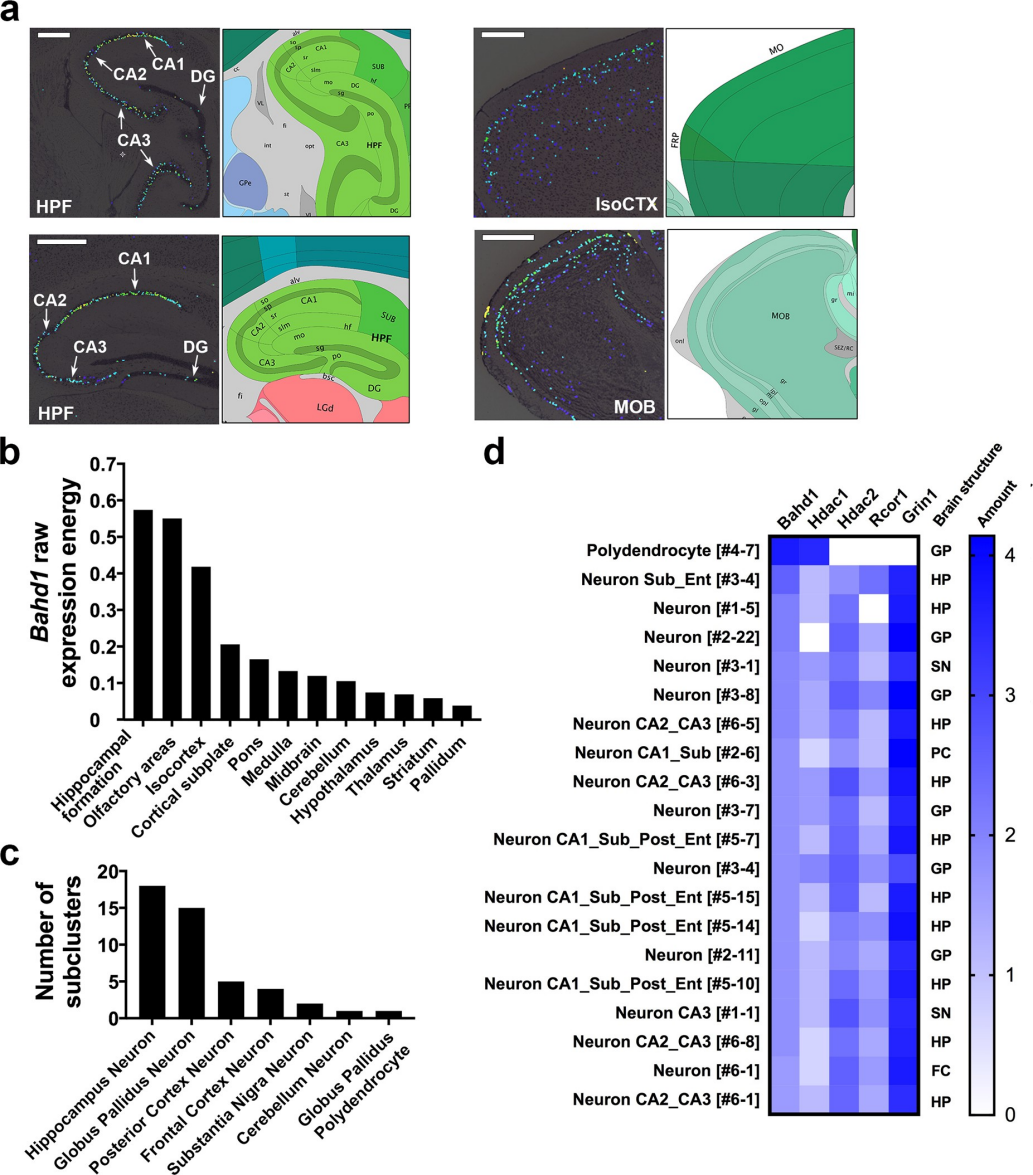
Expression of *Bahd1* in the mouse brain. **(a)** Examples of sagittal sections of brain regions with the highest *Bahd1* expression in the Allen Brain Atlas [22] (version 2019) showing the expression mask overlaid on the ISH (*Left*) and the corresponding atlas section (*Right*). Color code: *Bahd1* expression from the lowest (dark blue) to the highest level (yellow). Hippocampal Formation (HPF); Cornu Ammonis areas CA1, CA2, CA3; Dentate Gyrus, DG); Isocortex (IsoCTX); Olfactory Bulb (MOB). **(b**) Expression of *Bahd1* across the 12 major brain structures (from highest to lowest level). Reported values represent the average expression per region normalized to the average expression across the whole brain. (**c)** Number of brain cell subclusters with the highest *Bahd1* expression in the DropViz single‐cell gene expression database [24]. (**d)** Heat map showing the highest expression values for *Bahd1* in the cell subclusters in the DropViz database in comparison with that of *Hdac1*, *Hdac2* and *Rcor1*. *Grin1* was used as a control for a brain-specific gene. GP: Globus Pallidus; HP: Hippocampus; SN: Substantia Nigra; PC: Posterior Cortex; FC: Frontal Cortex.

We next determined the areas of the brain where *Bahd1* was most expressed using 3D-spatial gene expression data from the Allen Brain Atlas (ABA), which is the most comprehensive repository for *in situ* hybridization (ISH)-based gene data on expression in the adult mouse brain [22]. Analysis of ISH photomicrographs for the *Bahd1* probe (S5 Fig) revealed that across the brain, the intensity of the *Bahd1* signal was very low, except in three regions: the hippocampal formation (HPF), the isocortex and the olfactory regions (Fig 3A). An overview of the expression of *Bahd1* across 12 non-overlapping brain structures covering the entire brain confirmed the specific enrichment in these three regions (Fig 3B).

We then interrogated the mouse brain cell atlas accessible through the interactive online software DropViz [24] to profile the relative *Bahd1* mRNA expression in 262 cell types (690,000 individual cells) sampled from nine regions of the adult mouse brain. The RNA expression patterns enabled the classification of cells into transcriptionally distinct clusters and subclusters [24]. Forty-six subclusters belonging to seventeen clusters showed the highest expression of *Bahd1* (>1.5; *p* value<0.05) (S5 Table), of which the highest number (*i*.*e*., 18 subclusters) was in the hippocampus neurons (Fig 3C). Therefore, both the ISH and DropViz datasets showed consistent *Bahd1* expression in the hippocampus. The other subclusters encompassed neurons in the globus pallidus, posterior and frontal cortex, substantia nigra and cerebellum. Only one subcluster did not belong to a neuron type but to neuroglia in the Polydendrocyte.Tnr. Cyth3 [#4–7], which is a subcluster in the globus pallidus (S5 Table). Of note, the highest expression level of *Bahd1* was found in this subcluster (Fig 3D). We determined the expression levels of *Hdac1* and *Hdac2* in the same subclusters with *Bahd1* expression, as well as that of *Rcor1* (as an example of a gene encoding a subunit of another HDAC1/2 complex) and the brain-specific gene *Grin1* (Fig 3D and S5 Table). Interestingly, consistent with the fact that HDAC1 is mainly expressed in glia, whereas HDAC2 is expressed in neuronal cells [28], *Bahd1* was co-expressed with *Hdac1* in the Polydendrocyte [#4–7] subcluster, in which *Hdac2*, *Rcor1* and *Grin1* were not expressed. In contrast, there were much higher levels of *Hdac2* than *Hdac1* in neuron populations with the highest expression of *Bahd1* (Fig 3D).

**Fig 4:**
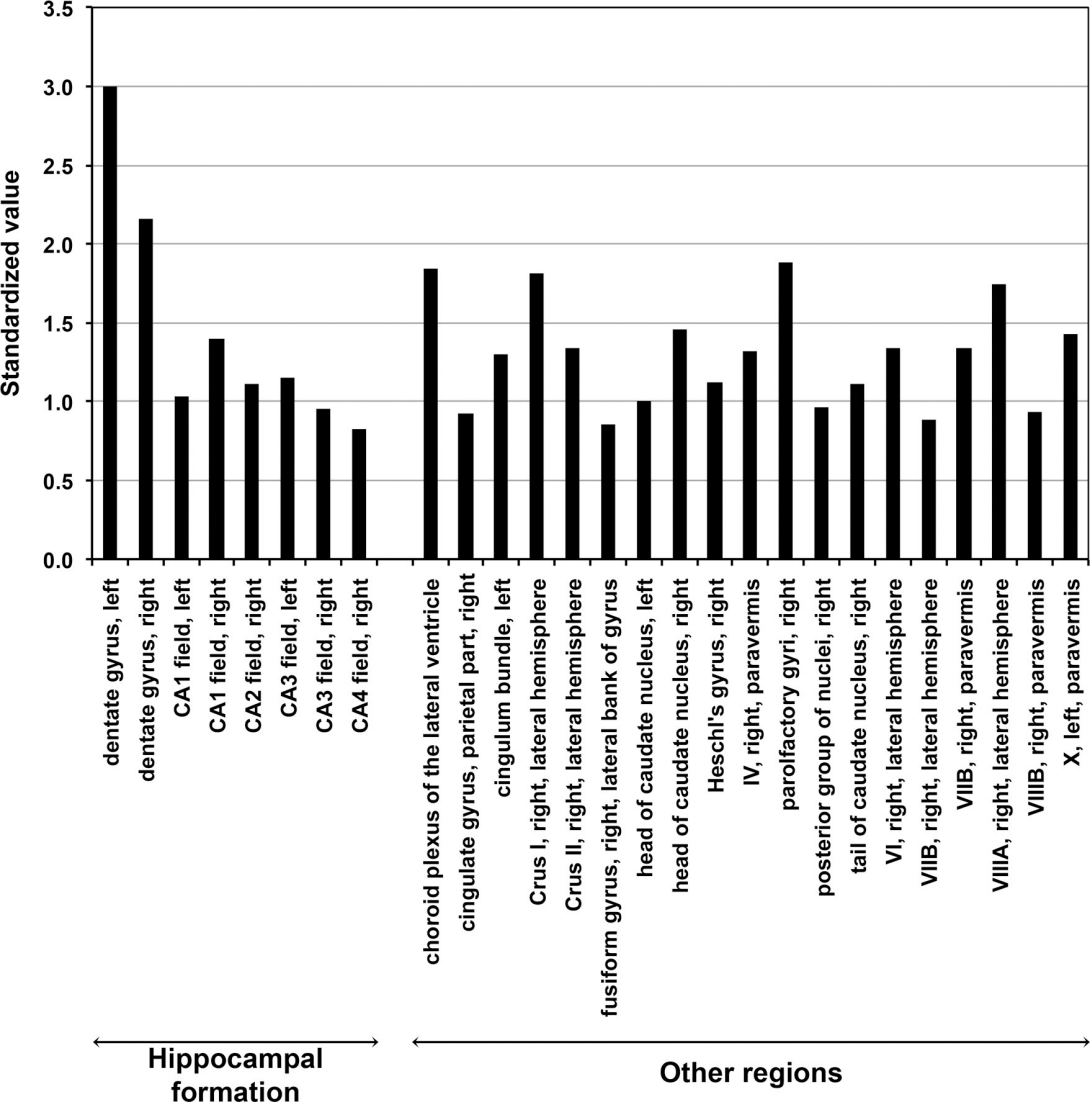
Expression of *BAHD1* in the human brain. Integrative analyze by Harmonizome database (https://amp.pharm.mssm.edu/Harmonizome/gene/BAHD1) of *BAHD1* mRNA expression profiles for six adult human brain tissue samples from the Allen Human Brain Atlas (http://human.brain-map.org)[23]. The 27 tissues with the highest expression of *BAHD1* relative to all brain tissues (about 300 brain structures) are shown. Standardized values are indicative of the relative strength of the functional associations and are related to empirical *p*-values, as “abs(standardized values) = -log10(*p*-value)”.

Finally, we explored the Allen Human Brain Atlas [23] (http://human.brain-map.org) to evaluate *BAHD1* expression in the human brain. This atlas includes microarray data for six adult human brain tissue samples spanning ~300 brain structures. The microarray data for *BAHD1* expression obtained with two independent probes showed the consistent enrichment of *Bahd1* expression in the hippocampal formation in all six brains (S6 Table). We also browsed the Harmonizome database [25], which provides an integrative analysis of these microarray data to highlight regions with the highest expression of a gene relative to that in other regions, regardless of the individual. The results showed that out of 27 brain tissues with the highest expression of *BAHD1*, 6 were in the hippocampus (dentate gyrus, CA1, CA2, CA3, CA4) (Fig 4). Altogether, these data suggest that BAHD1 could play a role in specific cell population, some of which in the hippocampal formation.

### Behavioral deficits in *Bahd1* haploinsufficient mice

**Fig 5:**
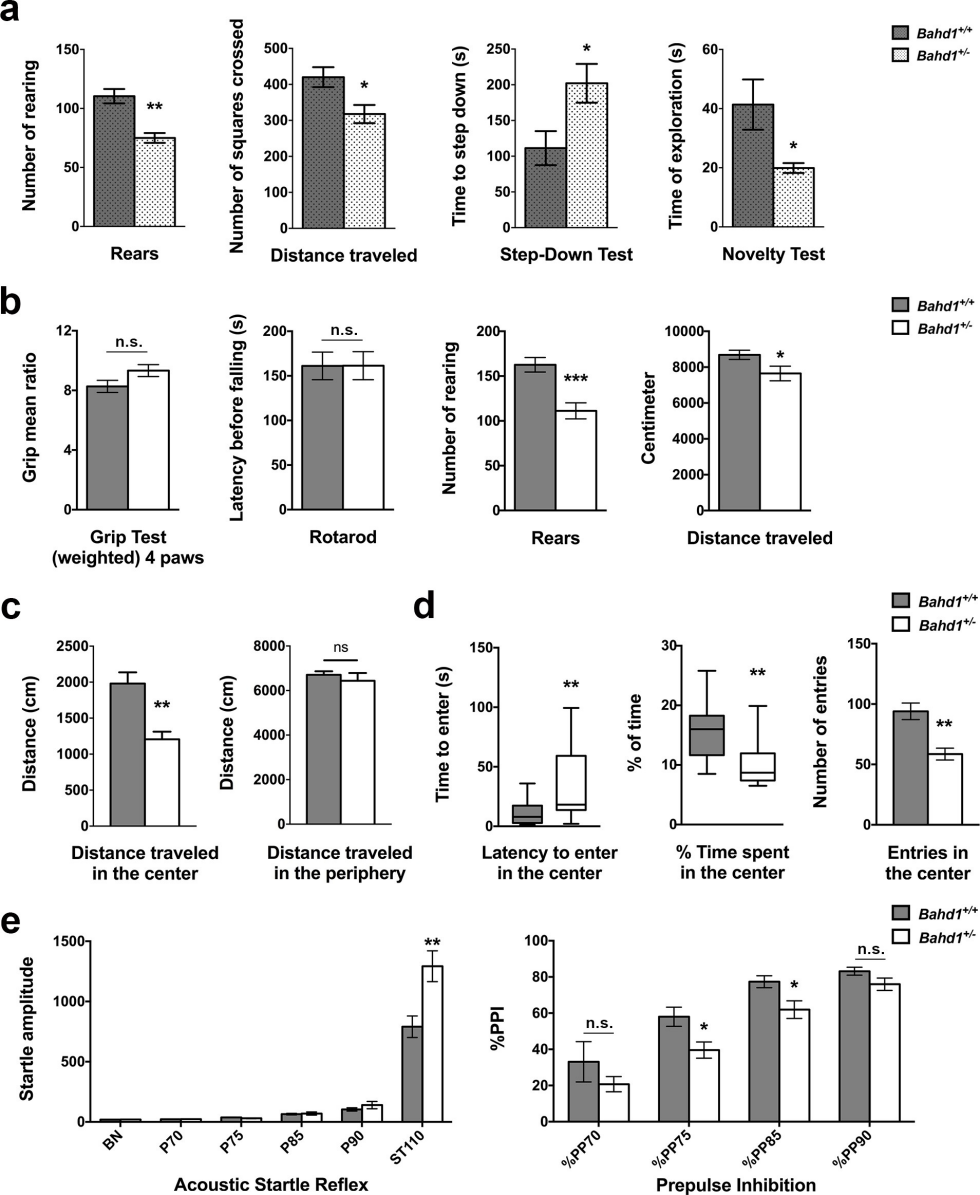
*Bahd1*^+/-^ mice show behavioral abnormalities. (**a**) Analysis of *Bahd1*-Het1 mice in comparison with their WT littermates. Number of rearings and distance travelled in the open field test; step-down test; novelty test. (**b-d**) Analysis of *Bahd1*-Het2 mice in comparison with their WT littermates. (**b**) Grip test; rotarod test; number of rearings and distance travelled in the open field test. (**c**) Distance travelled in the center versus periphery in the open field test. (**d**) Behavior of mice in relation to the center of the arena in the open field test. (**e**) Acoustic startle reflex (ASR) and prepulse inhibition (PPI) analysis (BN, background noise; P*x*, stimuli of *x*dB such as P70 = 70 dB; ST110, acoustic startle pulse of 110 dB; %PP*x*, prepulse of *x*dB followed by a pulse of 110 dB). Data are the mean ± SEM or median ± interquartile range, depending on the data normality, as assessed by the Shapiro-Walk normality test. Asterisks indicate significant differences (* *p*<0.05, ** *p*<0.01, *** *p*<0.001).

We aimed to evaluate whether changes in *Bahd1* expression levels in the brain could have an impact on behavior. The *Bahd1*-null mutation causes a significant level of perinatal death, which prevents the generation of mouse cohorts [6]. In contrast, heterozygous *Bahd1*^+/-^ mice [5], herein named “*Bahd1*-Het1”, survive and reproduce normally. Moreover, their general health, body weight, adipose tissue and basic sensory motor functions are not different from those of their WT littermates [6]. Therefore, this mouse line can be used to evaluate the effect of a partial deficit in BAHD1 on behavior. We first verified by RT-QPCR that the amount of *Bahd1* mRNA was decreased by approximately 50% in the brains of heterozygous animals relative to that in wild-type animals. The results were consistent with haploinsufficiency in *Bahd1* expression (S6 Fig). We then generated a cohort of ~10-week-old male mice, with seven animals per genotype. A composite neurological score generated by combining different tests (equilibrium, positioning, functional recovery, and tail suspension tests, S7 Table) confirmed the absence of the alteration of sensorimotor phenotypes in *Bahd1*-Het1 mice, suggesting that muscle strength and movement coordination were not affected by *Bahd1* haploinsufficiency. In the open field test, which is commonly used to assess both locomotor activity and anxiety-related responses by studying the exploratory behavior in an arena [29–31], there were no significant differences in the number of defecations or grooming between heterozygous and wild type individuals. However, the number of rearings and the distance travelled in the arena by the *Bahd1*-Het1 mice were decreased compared to those of WT littermates (Fig 5A). As this reduced explorative behavior could be linked to locomotion and/or anxiety, we specifically assessed anxiety by alternative tests. The step-down test records the time taken by a mouse to descend from an elevated platform. Anxious mice ordinarily remain on the platform for extended periods, whereas less anxious mice step off and explore [32]. The novelty test estimates the response to a potential threat by measuring the exploration time in the presence of an unknown object. Anxious mice will have the tendency to spend a decreased amount of time exploring the unknown object [33]. The *Bahd1*-Het1 mice took, on average, twice as long as WT littermates to get off the platform, and they spent half the time spent by WT mice in exploring the presented object (Fig 5A). Together, these results suggested that a decrease in *Bahd1* expression could increase anxiety.

To confirm these results and supplement them with the results of other tests, we used another cohort of heterozygous mice (C57BL/6N *Bahd1*^*tm1B(KOMP)Wts1*^, generated by the International Mouse Phenotyping Consortium), herein referred to as “*Bahd1*-Het2”. In these mice, the strategy used to inactivate *Bahd1* was slightly different from that used for the *Bahd1*-Het1 line (see details in the Supplementary information). Sixteen *Bahd1*-WT and nine *Bahd1*-Het2 male mice were studied at 9–10 weeks of age in a separate animal facility from that used for the *Bahd1*-Het1 cohort. A modified SHIRPA protocol (S8 Table) confirmed no significant difference between the *Bahd1* wild type and heterozygous genotypes in terms of general health status, various reflexes and basic sensory motor functions. The grip force and latency before falling off a rotarod were also not different (Fig 5B). In the open field test, *Bahd1*-Het2 mice showed a reduced number of rearings and a reduced distance travelled, as previously observed with *Bahd1*-Het1 mice. It should be noted that only the distance travelled in the center of the arena was reduced in the *Bahd1*-Het2 mice, while the distance travelled at the periphery was equivalent to that of the *Bahd1-*WT mice (Fig 5C). This tendency to stay close to the walls, called thigmotaxis, is an index of anxiety in mice [34]. The thigmotaxis value (i.e., the ratio of the distance travelled in the periphery to the total distance) for *Bahd1*-Het2 mice was increased by 7±2% (*p*<0.05) when compared to that of WT mice. *Bahd1*-Het2 mice also exhibited greater latency in entering the center of the arena and a decreased duration and number of entries in the center compared to WT mice (Fig 5D). Thus, in agreement with the results obtained with the first cohort, haploinsufficiency in *Bahd1* expression does not significantly impair muscular function or motor coordination but induces anxiety-like behavior.

The acoustic startle response (ASR) is also used to evaluate anxiety. In both laboratory animals and humans, a noise burst elicits a startle response and a higher startle magnitude is indicative of increased anxiety [30]. In addition, the ASR can be followed by a measure of the prepulse inhibition of the startle reflex (PPI), which evaluates sensorimotor gating (i.e. the ability of a sensory event to suppress a motor response). These tests assess the effectiveness of the neurological processes involved in filtering redundant or unnecessary sound stimuli. First, each mouse was subjected to increasingly loud stimuli (in decibels (dB)) that ranged from background noise (BN) to a level of 110 dB. ASR was evaluated according to the surprise amplitude of the animals. The startle reactivity for prepulses with lower intensities (BN to 90 dB) was comparable between *Bahd1*-Het2 and WT mice, indicating that *Bahd1*-Het2 mice were not deaf. More specific auditory evaluation using the auditory brainstem response (ABR) showed that *Bahd1*-Het2 and WT mice displayed comparable ABR thresholds for all of the acoustic stimuli frequencies used, ranging from 6 to 30 kHz (S7 Fig). On the other hand, at 110 dB mice *Bahd1*-Het2 mutants displayed significantly higher acoustic startle responses (SAs) compared to WT mice (Fig 5E), which could be interpreted as increased anxiety, in line with the results obtained in the open field test. When the startle pulse (110 dB) was preceded by prepulses with lower intensities (70 to 90 dB) that did not induce startle, the percentage of prepulse inhibition (% PPI) was lower in the *Bahd1*-Het2 mice than in the WT mice for the different prepulses. The differences were statistically significant for 75 or 85 dB prepulses (Fig 5E). This anomaly in the PPI could indicate degradation in the integration of the acoustic signal. PPI deficits have been observed in human subjects with schizophrenia and other neuropsychiatric disorders, as well as in established animal models of these disorders [35, 36]. Taken together, these results suggest that a deficit in BAHD1 impacts the behavior and could contribute to neuropsychiatric disorders.

## Discussion

BAHD1 is a subunit of a chromatin-remodeling complex, whose physiological functions are not well known. Here, we show that BAHD1 haploinsufficiency in the mouse led to behavioral alterations relevant to anxiety-like and neuropsychiatric disorders. We also provide evidence that the expression of the BAHD1 encoding gene is spatially restricted to small areas of the brain, including the hippocampal formation, cortical areas and the olfactory bulb. Interestingly, during the course of our study, the *BAHD1* human gene was identified as a disease locus correlated with mental disorder [37]. Together, these data support the hypothesis that BAHD1 dysfunction could contribute to behavioral disorders.

BAHD1 full deficiency causes changes in gene expression patterns at the whole brain level, suggesting that BAHD1 plays a role in chromatin-mediated gene expression in this organ. Approximately 2,300 protein-coding genes were deregulated in the brains of *Bahd1*-KO mice compared to those of wild type littermates, of which a majority was overexpressed, which is in agreement with the repressive function of BAHD1 [4, 6, 13]. The effect of the *Bahd1*-null mutation in the brain seems to occur at a postnatal stage, since we did not found any noticeable difference in the patterns of gene expression between the brains of WT and *Bahd1*-KO embryos at a late stage of embryonic development (E16.5). The main functions associated gene deregulations in adult *Bahd1*-KO brains were neuron development and function, behavior, learning and memory, and defense against pathogens (such as interferon-stimulated genes). We have previously identified a link between BAHD1 and interferon responses during the infection of human epithelial cells by the bacterial pathogen *L*. *monocytogenes* [5, 17]. *Listeria* causes CNS infections and interference with BAHD1 function by *Listeria* could be relevant in the brain.

However, we cannot draw many conclusions from whole-brain transcriptomic studies because they cannot distinguish between the direct and indirect effects of BAHD1 deficiency, nor can they determine in which brain territories and cells they occur. Advances in the exploration of BAHD1 functions require generating conditional *Bahd1*-KO mice to specifically knock-out *Bahd1* in regions of the brain and in specific cell populations in which *Bahd1* is specifically expressed. Here, we report that *Bahd1* expression is restricted to a few regions of the forebrain in mice, including the hippocampal formation, the isocortex and the olfactory bulb. Importantly, both ISH [22] and single-cell RNA-seq [24] expression studies in mice and transcriptomic analysis of the human brain showed consistent expression of the BAHD1 encoding gene in the hippocampal formation. This region is well known for its roles in spatial learning and memory, but a growing body of evidence supports the notion that it is also associated with aspects of emotionality and anxiety [38]. For instance, the hippocampal formation, by forming episodic representations of the emotional significance and interpretation of events, can influence the response of the amygdala when emotional stimuli are encountered [39]. Recent studies highlight that the hippocampus can be divided into multiple sub-regions with specific combinatorial gene expression patterns and interacting with different brain systems [40, 41]. This functional organization is proposed as the basis for the multifaceted role of the hippocampus in spatial navigation and behavior and, potentially, specific diseases. In this view, hippocampal dysfunction is implicated in a range of phenotypically diverse and mechanistically distinct disorders including learning disabilities and behavioral disorders, such as anxiety, depression and borderline personality disorders [42–44], as well as schizophrenia [45–47].

To explore the role of BAHD1 in behavior, we used heterozygous animals because they survived normally at birth and have no metabolic defects, unlike *Bahd1*-KO mice [6]. This model could mimic the effects of a partial deregulation of the *Bahd1* gene following disruption of homeostasis or upon the action of environmental factors. Different anxiety tests (i.e. the open field, novel object recognition, step-down and acoustic startle response) suggested that BAHD1 haploinsufficiency increased anxiety-related behavior. It also promoted a deficit in the PPI, which reflected the lack of the adaptation of their behavioral responses to successive sensory information. PPI deficits have been observed in various neuropsychiatric disorders, such as schizophrenia [35, 48, 49], obsessive-compulsive disorder [50], bipolar mania [51], panic disorder [52] and autism [53]. It is important to emphasize that Płoskia and collaborators recently reported the first genetic alteration of the human *BAHD1* gene and correlated this mutation with brain disorders [37]. They studied the genome of a girl born with a low birth weight (a phenotype also observed in *Bahd1*-KO mice [6]). Her physical and psychomotor development was nearly normal until the age of thirteen. Then, she experienced progressive intellectual deterioration with learning disabilities, anxiety, depression, social withdrawal and paranoid schizophrenia. Genome sequencing of this patient revealed a chromosomal translocation between the *RET* and *BAHD1* genes, leading to the fusion of the *RET* promoter with the coding part of *BAHD1* and the fusion of the *BAHD1* promoter with the coding part of *RET*. Since *RET* defects have not been associated with neurodevelopmental/psychiatric phenotypes, the authors suggested that it is likely that these phenotypes were caused by the deregulation of *BAHD1* expression. Based on our results in mice, we propose that in this patient, the *RET* promoter did not correctly drive the expression of *BAHD1* in the brain because it did not respond to the same signals as the native *BAHD1* promoter. Another remarkable phenotype in this patient that is relevant to our work is that she presented recurrent urinary tract infections since the age of ten months. Given the role of *BAHD1* in the expression of immunity genes, it is also possible that the recurrent infections in the patient were also part of the translocation phenotype.

**Fig 6:**
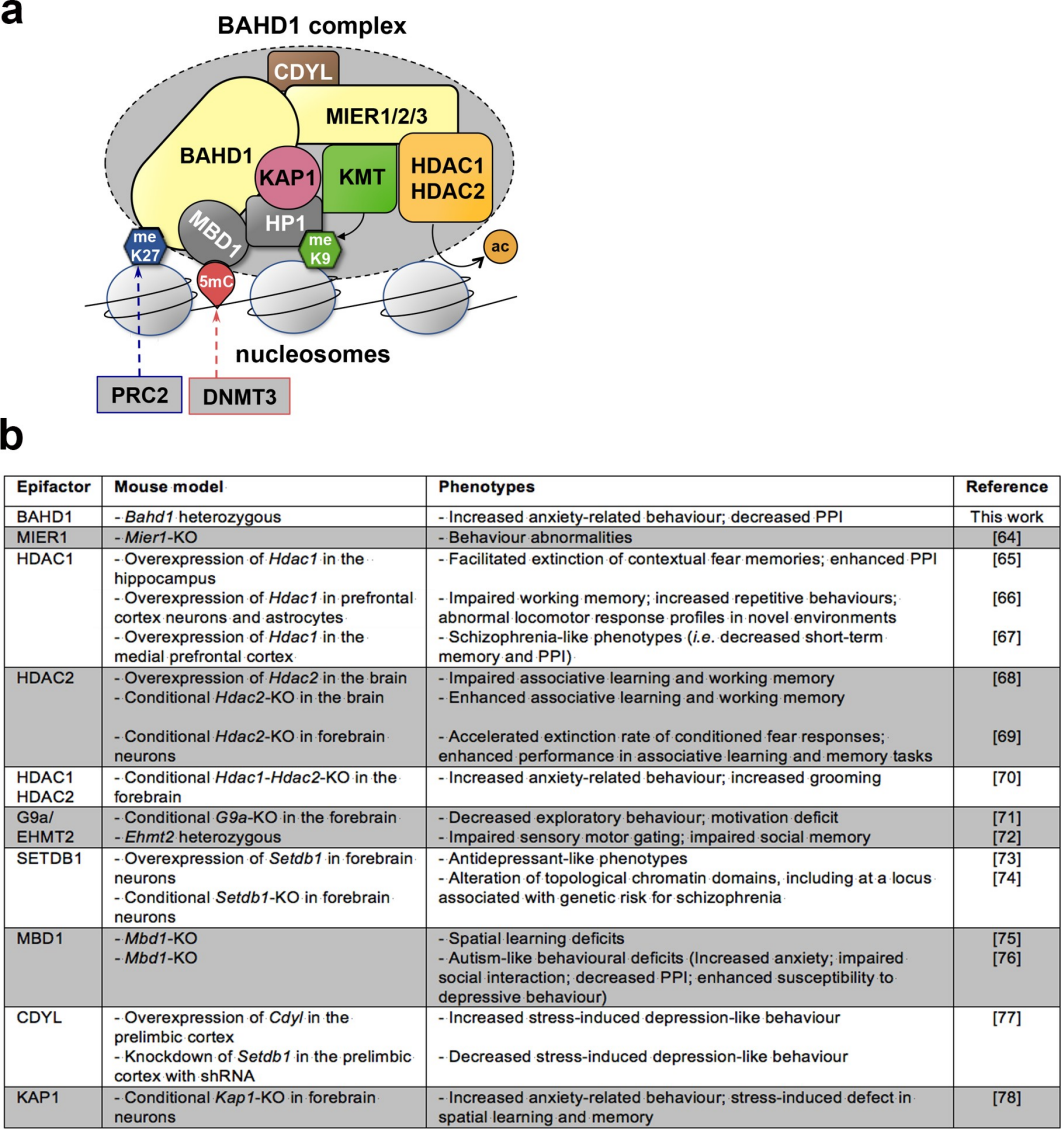
Relationship between the components of the BAHD1 chromatin-repressive complex and neurological disorders. (**a**) Schematic representation of the BAHD1 chromatin-repressive complex (KMT: histone lysine methyltransferase; meK9: histone H3 methylated at lysine 9; PRC2: Polycomb repressive complex 2; meK27: histone H3 methylated at lysine 27; DNMT3: DNA methyltransferase 3; 5mC: 5-methylcytosine; ac: removal of acetyl groups on histones by HDAC) (adapted from [6]). (**b**) Behavior-associated phenotypes in mice observed upon deficiency or overexpression of BAHD1-associated molecular partners and the references [59–73] for the relevant studies.

In recent years, the study of epifactors has shed light on the importance of epigenetic deregulation in neuropathologies and/or psychopathologies [54–58]. Interestingly, this includes several BAHD1-interacting partners, as summarized in Fig 6. The current model of the organization of the BAHD1 complex [6] proposes that the “duo” formed by the BAHD1 and MIER proteins constitutes a scaffold that connects chromatin-modification enzymes, such as KMTs (*i*.*e*., G9a, SETDB1, SUVH39H1) and HDAC1/2, to readers of epigenetic marks, such as HP1 and MBD1. BAHD1 itself is a reader of H3K27me3 [4, 14] (Fig 6A). Other proteins co-purified with BAHD1 were CDYL and KAP1 [6]. Deregulation of these epifactors induces significant alterations in brain-associated phenotypes in mice (Fig 6B), which are clearly linked to forebrain function deficits, including anxiety and cognitive defects, consistent with autism-like/schizophrenia-like phenotypes. Future studies are needed to determine how BAHD1 complexes assemble in specific brain cell types and particularly the subunits with which they associate. For instance, HDAC1 is mainly expressed in glia, whereas HDAC2 is expressed in neuronal cells [28]. Single-cell RNA-seq of murine brain cells suggested that BAHD1 is co-expressed with HDAC1 in a subcluster of polydendrocytes, which belong to the glia, whereas it is co-expressed with HDAC2 in different neurons in the hippocampus.

In conclusion, this study in mice and recent data from human pathology [37] lay the foundation for studying the contribution of BAHD1-dependent epigenetic regulation to brain function. By considering their phenotypes, *Bahd1*^+/-^ mice may provide a novel model for human mental disorders. However, future studies are needed to further characterize this model, including cognitive tests addressing the role of the hippocampus in spatial memory and tests examining the function of the olfactory bulb. There is growing suspicion that the disruption of the epigenetic machinery by exogenous molecules, such as chemical compounds, bacterial metabolites from the microbiota [74, 75] or virulence factors from invasive pathogens [76], contribute to complex diseases including psychiatric and neurodegenerative diseases [77, 78]. We suggest that disturbances in the function of the BAHD1 complex induced by bacteria, viruses, nutrients or endocrine disruptors, may be one of the mechanisms at work.

## Supporting information

S1 File (DOCX)

S1 Fig BAHD1 deficiency does not affect the general structure of the murine brain. Histological analysis of 17 month-old *Bahd1*^+/+^ and *Bahd1*^-/-^ mouse brain sections. Representative images of each genotype are showed. (**a**) Haematoxylin and eosin staining; (**b**) Periodic acid-Schiff (PAS) staining; (**c**) Luxol fast blue and cresyl violet staining of whole sagittal brain cuts.
(DOCX)

S2 Fig Transcriptome analysis of *Bahd1*^-/-^ brains (KO) in comparison with *Bahd1*^+/+^ (WT) brains. (**a, b**) Euclidian hierarchical clustering and Principal Component Analysis (PCA) of RNA-seq data. Cluster dendrograms are obtained from VST-transformed data. An euclidean distance is computed between samples and the dendrograms are built upon the Ward criterion. (**a**) RNA-seq data from half-brains of 17 month-old *Bahd1*-WT (WT1c, WT2c, WT3c) *vs*. *Bahd1*-KO (KO33c, KO56c, KO108c) mice. (**b**) RNA-seq data from whole brain of E16.5 embryos of *Bahd1*-WT (WT105c, WT112c, WT123c) *vs*. *Bahd1*-KO (KO71c, KO74c, KO78c) mice. (**c**) Analysis of DEGs between *Bahd1*-KO *vs*. *Bahd1*-WT embryonic brains, as in Fig 1A but without the KO78c outlier.
(DOCX)

S3 Fig *BAHD1* gene expression levels in human tissues in comparison with *GRIN1* and *RCOR1*. Data were extracted from Human Protein Atlas (HPA) available from v18.1.proteinatlas.org (www.proteinatlas.org). Specific image for *GRIN*, *BAHD1* and *RCOR1* and can be found at: https://www.proteinatlas.org/ENSG00000176884-GRIN1/tissue. https://www.proteinatlas.org/ENSG00000140320-BAHD1/tissue. https://www.proteinatlas.org/ENSG00000089902-RCOR1/tissue. 37 tissues have been analyzed by RNA-seq to estimate the transcript abundance of each protein-coding gene (for a total of 172 tissue samples) [11]. HPA RNA-seq tissue data is reported as mean TPM (protein-coding transcripts per million), corresponding to mean values of the different individual samples from each tissue. Color-coding is based on tissue groups. For tissue type, the average TPM value for replicate samples was used as abundance score. The threshold level to detect presence of a transcript for a particular gene was set to ≥ 1 TPM. *GRIN1* belongs to “Tissue enriched” category of genes (expression in one tissue at least five-fold higher than all other tissues/cell lines) and is enriched in the brain. *BAHD1* and *RCOR1* belong to the “Expressed in all tissues” category of genes (≥ 1 TPM in all tissues/cell lines).
(DOCX)

S4 Fig Expression profiling of *Grin1*, *Bahd1* and *Rcor1* in mouse tissues. RNA profiling data sets were generated by the Mouse ENCODE project [12] from C57BL/6 mice tissues of two biological replicates. Graphs are adapted from those released in the NCBI database (https://www.ncbi.nlm.nih.gov) for *Grin1* (Gene ID 14810), *Bahd1* (Gene ID: 228536) and *Rcor1* (Gene ID: 217864). Histograms represent RNA-seq results reported as RPKM (Reads Per Kilobase Million). Adult tissues were taken from 8-week old littermates. Embryonic tissues of the Central nervous system (CNS) were taken from stage E11.5, E14 and E18 littermates.
(DOCX)

S5 Fig *BAHD1* expression in a set of sagittal sections from the allen mouse brain atlas. Masks derived from the ISH results (*Left*), and the corresponding atlas section (*Right*) are shown. The regions with the highest *BAHD1* expression are pointed with arrows *(*Hippocampal Formation (HPF), Olfactory areas (OLF) and Isocortex). (Adapted from https://mouse.brain-map.org) [13].
(DOCX)

S6 Fig Relative expression levels of *Bahd1* mRNA in *Bahd1*-HET1 in comparison with *Bahd1*-WT brains by RT-qPCR. Histograms show the relative *Bahd1* expression in 7 WT and 7 HET mice (relative to WT1) (*Left*), and mean ± S.E.M of *Bahd1* expression in all HET relative to all WT mice (*Right*) (**** *p*<0 .0001).
(DOCX)

S7 Fig Audiograms of *Bahd1*-WT and *Bahd1*-Het2 mice. The auditory brainstem response test determines hearing sensitivity using evoked potential recordings in anaesthetized mice. ABR thresholds (decibels of sound pressure level, dB SPL) were recorded to the following frequencies and intensities of stimuli; 6kHz (20-85dB SPL), 12kHz (0-70dB SPL), 18kHz (0-70dB SPL), 24kHz (10-70dB SPL) and 30kHz (20-85dB SPL), presented in 5dB intervals. The *Bahd1*-Het2 mice show comparable ABR when compared to WT mice.
(DOCX)

S1 Table Differentially-expressed genes in *Bahd1*-KO *vs*. *Bahd1*-WT mice half-brains (*p* <0.05; fold change ≥2 or ≤-2). (XLSX)

S2 Table Predicted biological functions and diseases associated with up-regulated BAHD1-diff genes by IPA (*p*<0.0005). Biological functions and diseases associated with genes up-regulated in *Bahd1*-KO vs. *Bahd1*-WT brains. The *p* value is the probability that the gene changes are related to a particular function or disease just by chance. Functions and Diseases are ranked according to the p-value (for categories ≥10 genes). Boxes in yellow and green highlight the sub-categories shown in Table 1.
(XLSX)

S3 Table Predicted biological functions and diseases associated with down-regulated BAHD1-diff genes by IPA (*p*<0.0005). Biological functions and diseases associated with genes up-regulated in *Bahd1*-KO vs. *Bahd1*-WT brains. The *p* value is the probability that the gene changes are related to a particular function or disease just by chance. Functions and Diseases are ranked according to the *p*-value (for categories ≥10 genes). Blue boxes highlight the sub-categories shown in Table 1.
(XLSX)

S4 Table List of down-regulated BAHD1-diff genes involved in immunity, inflammation and/or host defenses. The following table recapitulates the list of protein-coding genes down-regulated in *Bahd1*-KO compared to *Bahd1*-WT brains, which are involved in host defense to infections, immunity and/or inflammation. (after Benjamini Hochberg *p*-value adjustment (*p*<0.05) and down-regulation of at least 2 fold).
(XLSX)

S5 Table DropViz brain cell clusters and subclusters with the highest expression of *Bahd1*. DropViz (http://dropviz.org) provides the Drop-seq data of single-cell RNA sequencing of 690,000 cells sampled from nine regions of the adult mouse brain. The cells' patterns of RNA expression enables a classification into transcriptionally distinct groups of cells (clusters and subclusters). The table presents the subclusters with the highest *Bahd1* amounts (>1.5; *p*<0.05). Values for *Hdac1*, *Hdac2*, *Rcor1* and *Grin1* amounts in these subclusters are indicated. Subclusters in the hippocampus are highlighted in grey.
(XLSX)

S6 Table *BAHD1* expression in the human brain. Quantitative microarray gene expression data for BAHD1 was obtained from the Allen Human Brain Atlas resource.The profiles of the six donors is indicated on the headlines and additionnal informations can be found at http://human.brain-map.org. Each table presents the top 10 regions with the highest BAHD1 transcript levels for two probes (probe1 and probe2). The hippocampal formation is highlighted in grey.
(XLSX)

S7 Table Detailed results of the neurological examination of *Bahd1*-Het1 and -WT mice (cohort 1). A battery of 10 tests was designed to evaluate equilibrium, muscle strength, several reflexes and sensorimotor functions (as detailed in the supplementary information).
(XLSX)

S8 Table Detailed results of the modified SHIRPA on *Bahd1*-Het2 and WT mice (cohort 2). (XLSX)
