# Correction: BAHD1 haploinsufficiency results in anxiety-like phenotypes in male mice

**DOI:** 10.1371/journal.pone.0320947

**Published:** 2025-03-21

**Authors:** The PLOS One Staff

The references in Fig 6 are incorrect. The authors have provided a corrected version here. The publisher apologizes for the error.

**Fig 6:**
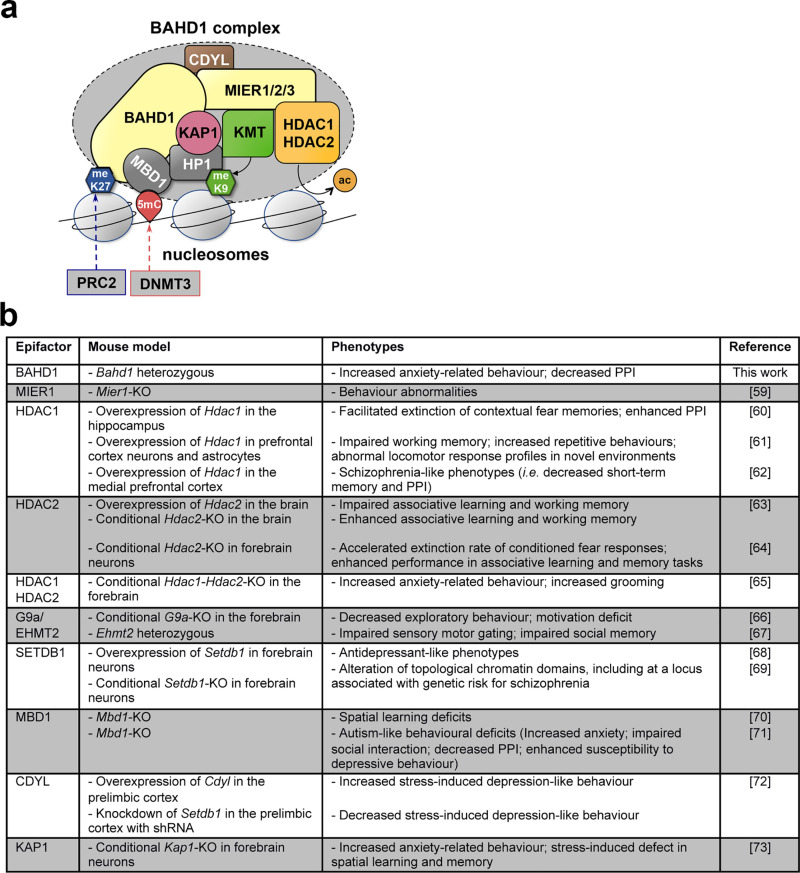
Relationship between the components of the BAHD1 chromatin-repressive complex and neurological disorders. **(a)** Schematic representation of the BAHD1 chromatin-repressive complex (KMT: histone lysine methyltransferase; meK9: histone H3 methylated at lysine 9; PRC2: Polycomb repressive complex 2; meK27: histone H3 methylated at lysine 27; DNMT3: DNA methyltransferase 3; 5mC: 5-methylcytosine; ac: removal of acetyl groups on histones by HDAC) (adapted from [6]). **(b)** Behavior-associated phenotypes in mice observed upon deficiency or overexpression of BAHD1-associated molecular partners and the references [59–73] for the relevant studies.

## References

[1] Pourpre R, Naudon L, Meziane H, Lakisic G, Jouneau L, Varet H, et al. BAHD1 haploinsufficiency results in anxiety-like phenotypes in male mice. PLoS One. 2020;15(5): e0232789. doi: 10.1371/journal.pone.0232789 32407325 PMC7224496

