# Supplementary material for: BAHD1 haploinsufficiency results in anxiety-like phenotypes in male mice
Source: PLoS One. 2020 May 14;15(5):e0232789. doi: 10.1371/journal.pone.0232789 (PMC7224496; doi:10.1371/journal.pone.0232789)
Supplement: S1 File — (DOCX) [file pone.0232789.s001.docx]

**BAHD1 deficiency alters the brain transcriptome and behaviour of male mice**

Renaud Pourpre^1^, Laurent Naudon^1,2#^, Hamid Meziane^3#^, Goran Lakisic^1^, Luc Jouneau^4^, Hugo Varet^5,6^, Rachel Legendre^5,6^, Olivia Wendling^3^, Mohammed Selloum^3^, Caroline Proux^6^, Jean-Yves Coppée^6^, Yann Herault^3^ and Hélène Bierne^1*^

^1^ Université Paris-Saclay, INRAE, AgroParisTech, Micalis Institute, Jouy-en-Josas, France

^2^ Université Paris-Saclay, CNRS, INRAE, AgroParisTech, Micalis Institute, Jouy-en-Josas, France.

^3^ Université de Strasbourg, CNRS, INSERM, Institut Clinique de la Souris-ICS, PHENOMIN, Illkirch, France.

^4^ Université Paris-Saclay, INRAE, Virologie et Immunologie Moléculaires, Jouy-en-Josas, France.

^5^Institut Pasteur - Bioinformatics and Biostatistics Hub - C3BI, USR 3756 IP CNRS, Paris, France.

^6^Institut Pasteur, Transcriptome and Epigenome Platform, Biomics Pole, C2RT Paris, France.

^7^Université de Strasbourg, CNRS, INSERM, Institut de Génétique Biologie Moléculaire et Cellulaire (IGBMC), UMR7104, U1268, Illkirch, France

**1. Supplemental Methods**

**RNA-seq data: read mapping, gene counting, normalization and statistical analyzes.**

Reads were cleaned of adapter sequences and low-quality sequences were removed using Cutadapt (v1.11). STAR version 2.5.0a, with default parameters, was used for alignment on the reference genome (from Ensembl-92 GRCm38 assembly). Libraries were pooled in equimolar proportions and sequenced on a paired-end 50-34 run, on an Illumina NextSeq500 instrument, using a NextSeq 500/550 High Output Kit v2 (75 cycles). Demultiplexing was done (bcl2fastq2 V2.2.18.12) and adapters removed (Cutadapt v1.15). Only reads longer than 10pb were kept for analysis. Tophat2 (v 2.1.1), with “-N 2 --read-edit-dist 2 --b2-sensitive--no-coverage-search” parameters was used for alignment on the reference genome (from Ensembl-92 GRCm38 assembly). Genes were counted using featureCounts version 1.4.6-p3 from Subreads package (parameters: -t exon -g gene_id -s 1). For all transcriptomic analysis, data were evaluated through principal component analysis and hierarchical clustering after transformation of the count data using RLOG function (DESeq2). Dendograms were built using Euclidian distance function and Ward criterion as linkage function. Normalization and differential analysis were carried out using the DESeq2, with a pre-filter step to remove genes with low counts (sum of all samples counts <10). Gene types were assessed on Ensembl-92 GRCm38. We adjusted the reported *p*-values by performing the Benjamini-Hochberg procedure to control the False Discovery Rate (FDR).

**Generation of *Bahd1*-Het2 mice.**

The *Bahd1* ^tm2b (KOMP) Wtsi^ line, referred to as “*Bahd1*-Het2”, was established at the MCI/ICS (Mouse Clinical Institute, Institut Clinique de la Souris, Illkirch, France; <http://www-mci.u-strasbg.fr>) in the framework of the International Mouse Phenotyping Consortium (IMPC [1]). The strategy involved the design of a conditional ready tm1a allele in ES cells. Briefly, exon 2 of the *Bahd1* allele (Ensembl Gene ID: ENSMUSG00000040007) was flanked by two loxP sites, a FRT-lacZ cassette and a floxed neomycin cassette. C57BL/6N mice generated with the *tm1a* allele were crossed with a Cre driver line to both eliminate exon 2 and remove the selection cassette, generating the tm1b null allele. The *tm1b* allele was genotyped as in [2].


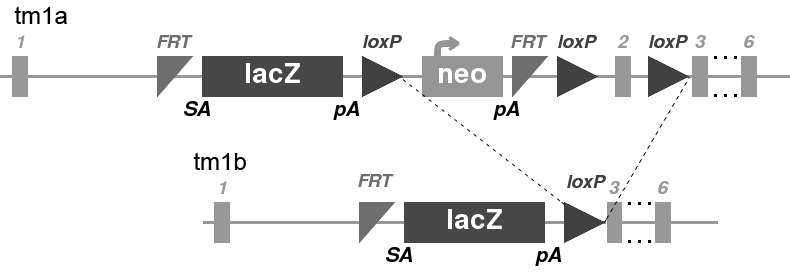


Design of the Bahd1 ^tm2b (KOMP) Wtsi^ heterozygous mouse.

**Quantitative RT-PCR primers**

| **Mouse transcript** | **Forward sequence** | **Reverse sequence** | **Reference** |
| --- | --- | --- | --- |
| *Ywhaz* | TAAATGGTCTGTCACCGTCT | GGAAATACTCGGTAGGGTGT | [3] |
| *Hprt* | Qiagen PPM03559E | Qiagen PPM03559E | Qiagen PPM03559E |
| *Bahd1* | Qiagen PPM40834A | Qiagen PPM40834A | Qiagen PPM40834A |
| *Lyz1* | AATGTGCAAAGAGGGTGGTGA | GGGAAAGCGAGGAAGTGTGAC | This work |
| *Sftpa1* | AATGGGAGTCCTCAGCTTG | ACTGACTGCCCATTGGTG | [4] |
| *Btla* | CAGAATTGTCTCCGGCTTTA | AGGGTGCCTGACTTTCTCTT | This work  (realtimeprimers.com) |
| *Ccl9* | CCCTCTCCTTCCTCATTCTTACA | AGTCTTGAAAGCCCATGTGAAA | [5] |
| *Ifi204* | GGGGAGTGGAAAATGGCACA | GCACCATCACTTGTTTGGGA | [6] |
| *Mndal* | AGGCATCCTGGAGATCAATG | GGACTGGCATTTGCATTTCT | This work  (realtimeprimers.com) |

**Behavioral tests and statistics**

Behavior tests were conducted at different times and the mice were allowed to rest for one to three days between each test. In addition, the order of the tests was carefully established, from least stressful to most stressful.

**Cohort1 (i.e. Bahd1-Het1 and WT littermates)**

***Neurological examination.*** Neurological examination of cohort1 was performed as described in [7]. A battery of 10 tests was designed to evaluate equilibrium, muscle strength and several reflexes and to assess the sensorimotor functions and general capacities of the mice (Supplementary Table S8). Equilibrium was assessed by the ability of the mouse to grab a horizontal rod with one to four paws (1 point per paw), and to turn around on a sloped plane in less than 30 seconds (0-15 seconds: 2 points; 15-30 seconds: 1 point; >30 seconds: 0). Gripping was evaluated by flexion of each paw after contact of a metal rod with the inner surface of the paw and resistance to removal of the rod (1 point per paw). The visual placement was evaluated by suspending the mouse by its tail and then gently approaching the edge of a table and testing its ability to immediately press its two front paws against the edge of the table (if successful, 1 point). To evaluate the loss of support, the mouse was held at the edge of a table and a paw was brought away of the table. To pass the test, the mouse had to immediately place it back on the edge of the table. This was repeated for each paw (1 point per paw). To measure the righting reflex, two tests were used. In the first, the mouse was held on its back, once released, to pass the test it had to immediately straighten up on these 4 paws. In the second test, the mouse was held on its back and released from a height of 20 cm. To pass the test, it had to fall back on its 4 paws. The quality of the gait was observed on a flat surface (for a normal gait: 2 points; if the gait was altered: 1 point; if the mouse was immobile: 0). During a tail suspension test, the mouse had to extend the front paws (1 point per paw in extension) and twist the chest to move up right or left (1 point per side). The composite neurological score for each mouse was obtained by adding the scores of each test.

***Open-field behaviour.*** Mice were placed in a rectangular arena (19 cm high, 21 cm large, 37 cm long), which floor is divided into 5 x 7 squares. The open field was placed in a room homogeneously illuminated at 70 lx. Each mouse was placed in a corner of the testing field and allowed to explore freely for 10 min. The numbers of crossed squares, rears, grooming and defecations, were monitored and recorded in the 10 min testing period.

***Novelty test.*** This test adapted from [8] was performed in the open field arena described above. An unknown object was placed 7.5 cm from one of the short side of the rectangular arena. Each mouse was placed facing the opposite wall. The time spent exploring the object (*i.e* the nose directed towards the object at a distance of less than 2 cm) and the distance travelled in the arena were monitored for 10 min.

***Step-down test.*** This test is adapted from [9]. It was designed to assess the readiness of the mouse to escape from an elevated place by stepping-down onto an horizontal surface, here a small platform (4 cm high, 13 cm long, 9.5 cm large) made of Lego®. Each mouse was gently put on the platform and the latency to step down with all four paws was recorded during a 3 trials of 5 min each with a 1 min interval. The platform was cleaned with a wet cloth after completion of testing of each mouse. The average latency obtained from the 3 trials was used to evaluate the performance.

**Cohort2 (i.e. Bahd1-Het2 and WT littermates)**

***Neurological examination.*** General health and basic sensory motor functions of *Bahd1*-Het2 mice and WT littermates were assessed using a modified SHIRPA protocol: (IMPReSS, http://www.mousephenotype.org/impress/ProcedureInfo?action=list&procID=797).

This analysis provides an overview of physical appearance, body weight, neurological reflexes and sensory abilities (Supplementary Table S9).

***Rotarod test.*** This test measures the ability of an animal to maintain balance on a rotating rod (Bioseb, Chaville, France). Mice were given three testing trials during which the rotation speed accelerated from 4 to 40 rpm in 5 min. Trials were separated by 5-10 min interval. The average latency was used as index of motor coordination performance.

***Grip test.*** This test measures the maximal muscle strength (g) using an isometric dynamometer connected to a grid (Bioseb). Mice were allowed to grip the grid with all its paws then they were pulled backwards until they released it. Each mouse was submitted to three consecutive trials immediately after the modified SHIRPA procedure. The maximal strength developed by the mouse before releasing the grid was recorded and the average value of the three trials adjusted to body weight.

***Open-field behaviour***. Mice were tested in automated open fields (Panlab, Barcelona, Spain), each virtually divided into central and peripheral regions. The open fields were placed in a room homogeneously illuminated at 150 lx. Each mouse was placed in the periphery of the open field and allowed to explore freely the apparatus for 20 min, with the experimenter out of the animal’s sight. The distance travelled, the number of rears and time spent in the central and peripheral regions were recorded over the test session. Thigmotaxis value was calculated by the ratio of the distance travelled in the periphery to the total distance covered expressed as a percentage [10]. The latency to enter was assessed by measuring the time that takes a mouse to enter in the center of the arena for the first time. The number of entries in center was also measured.

***Acoustic startle reflex (ASR) and prepulse inhibition (PPI) tests****.* ASR and PPI were assessed in a single session using standard startle chambers (SR-Lab Startle Response System, San Diego Instruments, USA). Ten different trial type were used: acoustic startle pulse alone (110-db), eight different prepulse trials in which either 70, 75, 85 or 90-dB stimuli were presented alone or preceded the pulse, and finally one trial (NOSTIM) in which only the background noise (65 dB) was presented to measure the baseline movement in the Plexiglas cylinder. In the startle pulse or prepulse alone trials, the startle reactivity was analysed, and in the prepulse plus startle trials the amount of PPI was measured and expressed as percentage of the basal startle response.

***Auditory brainstem responses (ABR).*** Auditory brainstem response test determines hearing sensitivity using evoked potential recordings in anaesthetized mice. The ABRs are recorded using an electrophysiological station composed of different items from Tucker-Davis Technologies (Alachua, FL, USA). The mouse is anesthetized with a mixture of Ketamine-Xylazine. It is then placed in a sound-attenuating chamber facing a loud-speaker and recording electrodes appropriately placed on the skull. ABRs are recorded to different acoustic stimuli. ABRs are first recorded to clicks (white noise) (10 µs duration, positive transient) presented from 0-85 dB SPL in 5dB steps, presented 256 times at 42.6/sec. ABRs are then recorded to the following frequencies and intensities of stimuli; 6kHz (20-85dB SPL), 12kHz (0-70dB SPL), 18kHz (0-70dB SPL), 24kHz (10-70dB SPL) and 30kHz (20-85dB SPL), presented in 5dB intervals. (Tone pips are 5ms in duration, with a 1ms rise/fall time, presented 256 times at 42.6/sec). At the end of recording, a final recording for clicks is performed. After recovery from anesthesia, mice are returned to their home cages.

**Statistics.**

For both cohorts, normally distributed data with equal group variances were expressed as means ± standard errors of the means (sem) and analysed with a student-test. Non-normally distributed data, or with unequal group variances, were expressed as medians and interquartile ranges, and analysed with the Mann-Whitney test. The level of significance was set at p<0.05. Significant differences are represented by asterisk (* p<0.05, ** p<0.01, *** p<0.001). Calculations were performed with the GraphPad Prism software.

**2. Supplementary References**

1. Brown SD, Moore MW. The International Mouse Phenotyping Consortium: past and future perspectives on mouse phenotyping. Mamm Genome. 2012;23(9-10):632-40. doi: 10.1007/s00335-012-9427-x.

2. Lebreton A, Lakisic G, Job V, Fritsch L, Tham TN, Camejo A, et al. A bacterial protein targets the BAHD1 chromatin complex to stimulate type III interferon response. Science. 2011;331(6022):1319-21. doi: 10.1126/science.1200120.

3. Lakisic G, Lebreton A, Pourpre R, Wendling O, Libertini E, Radford EJ, et al. Role of the BAHD1 Chromatin-Repressive Complex in Placental Development and Regulation of Steroid Metabolism. PLoS Genet. 2016;12(3):e1005898. doi: 10.1371/journal.pgen.1005898.

4. Bird AD, Flecknoe SJ, Tan KH, Olsson PF, Antony N, Mantamadiotis T, et al. cAMP response element binding protein is required for differentiation of respiratory epithelium during murine development. PLoS One. 2011;6(3):e17843. doi: 10.1371/journal.pone.0017843.

5. Arima Y, Harada M, Kamimura D, Park JH, Kawano F, Yull FE, et al. Regional neural activation defines a gateway for autoreactive T cells to cross the blood-brain barrier. Cell. 2012;148(3):447-57. doi: 10.1016/j.cell.2012.01.022.

6. Stavrou S, Blouch K, Kotla S, Bass A, Ross SR. Nucleic acid recognition orchestrates the anti-viral response to retroviruses. Cell Host Microbe. 2015;17(4):478-88. doi: 10.1016/j.chom.2015.02.021.

7. Capdeville C, Plotkine M, Boulu RG. [Methods for evaluating the neurologic deficit induced by transient cerebral ischemia in the unanesthetized rat]. J Pharmacol. 1984;15(2):231-7.

8. Ennaceur A, Michalikova S, Bradford A, Ahmed S. Detailed analysis of the behavior of Lister and Wistar rats in anxiety, object recognition and object location tasks. Behav Brain Res. 2005;159(2):247-66. doi: 10.1016/j.bbr.2004.11.006.

9. Anisman H, Hayley S, Kelly O, Borowski T, Merali Z. Psychogenic, neurogenic, and systemic stressor effects on plasma corticosterone and behavior: mouse strain-dependent outcomes. Behav Neurosci. 2001;115(2):443-54.

10. Simon P, Dupuis R, Costentin J. Thigmotaxis as an index of anxiety in mice. Influence of dopaminergic transmissions. Behav Brain Res. 1994;61(1):59-64. doi: 10.1016/0166-4328(94)90008-6.

11. Uhlen M, Fagerberg L, Hallstrom BM, Lindskog C, Oksvold P, Mardinoglu A, et al. Proteomics. Tissue-based map of the human proteome. Science. 2015;347(6220):1260419. doi: 10.1126/science.1260419. PubMed PMID: 25613900.

12. Yue F, Cheng Y, Breschi A, Vierstra J, Wu W, Ryba T, et al. A comparative encyclopedia of DNA elements in the mouse genome. Nature. 2014;515(7527):355-64. doi: 10.1038/nature13992.

13. Lein ES, Hawrylycz MJ, Ao N, Ayres M, Bensinger A, Bernard A, et al. Genome-wide atlas of gene expression in the adult mouse brain. Nature. 2007;445(7124):168-76. doi: 10.1038/nature05453.
