# Supplementary material for: BAHD1 haploinsufficiency results in anxiety-like phenotypes in male mice
Source: PLoS One. 2020 May 14;15(5):e0232789. doi: 10.1371/journal.pone.0232789 (PMC7224496; doi:10.1371/journal.pone.0232789)
Supplement: S1 Fig — Histological analysis of 17 month-old Bahd1+/+ and Bahd1-/- mouse brain sections. Representative images of each genotype are showed. (a) Haematoxylin and eosin staining; (b) Periodic acid-Schiff (PAS) staining; (c) Luxol fast blue and cresyl violet staining of whole sagittal brain cuts. (DOCX) [file pone.0232789.s002.docx]

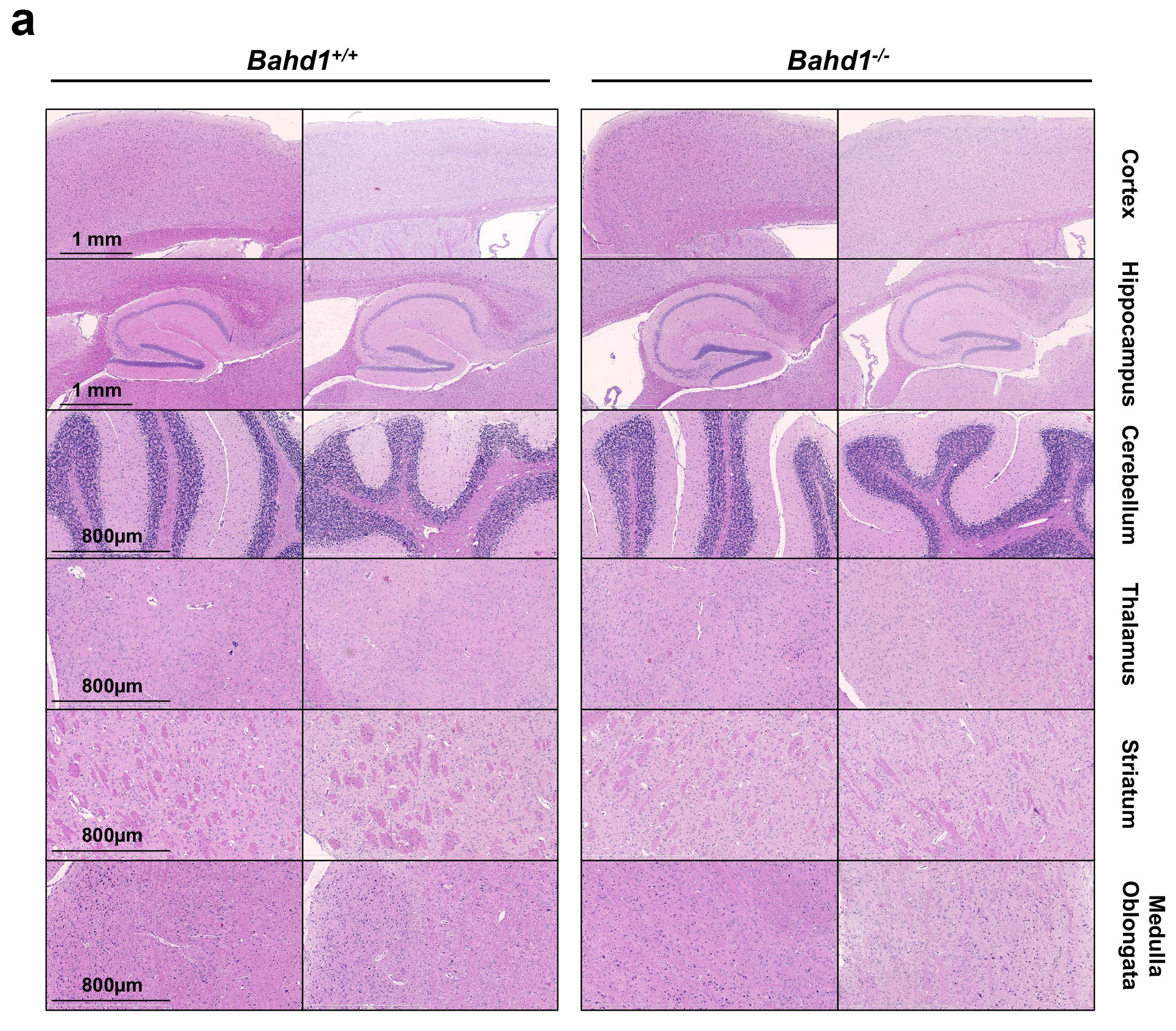


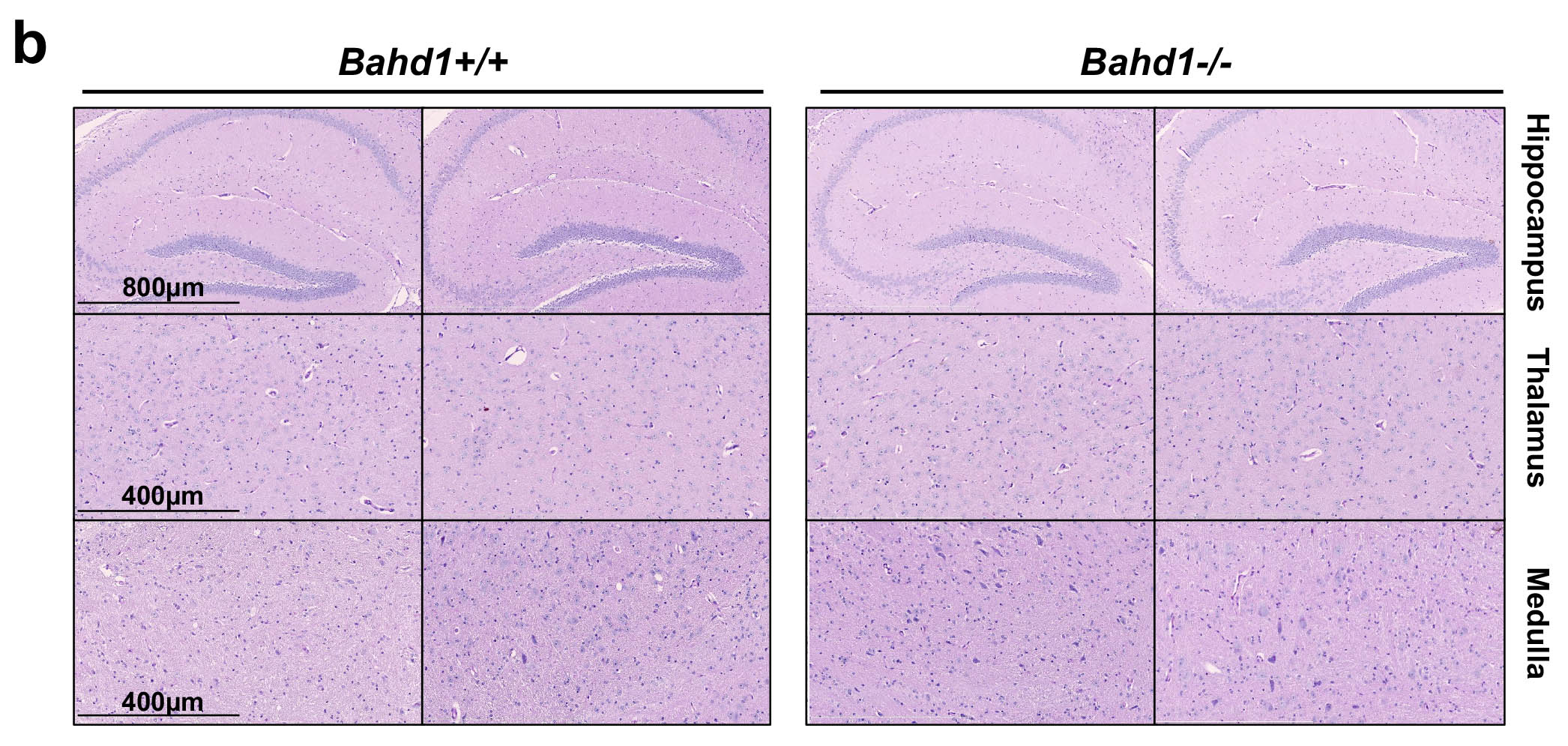


**
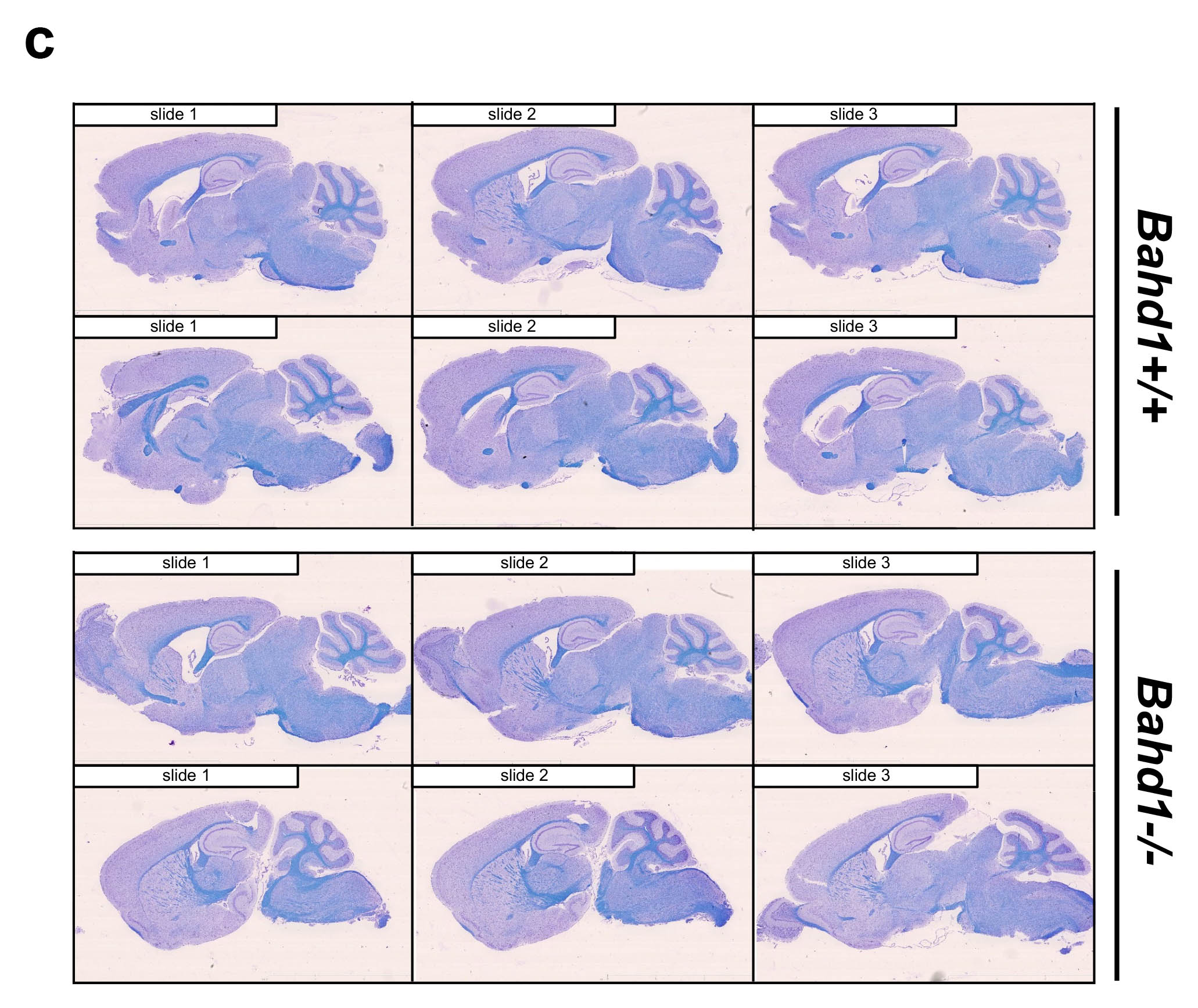
**

**S1 Fig**. **BAHD1** **deficiency does not affect the general structure of the murine brain**. Histological analysis of 17 month-old *Bahd1*^+/+^ and *Bahd1*^-/-^ mouse brain sections. Representative images of each genotype are showed. (**a**) Haematoxylin and eosin staining; (**b**) Periodic acid-Schiff (PAS) staining; (**c**) Luxol fast blue and cresyl violet staining of whole sagittal brain cuts.
