# Supplementary material for: BAHD1 haploinsufficiency results in anxiety-like phenotypes in male mice
Source: PLoS One. 2020 May 14;15(5):e0232789. doi: 10.1371/journal.pone.0232789 (PMC7224496; doi:10.1371/journal.pone.0232789)
Supplement: S2 Fig — (a, b) Euclidian hierarchical clustering and Principal Component Analysis (PCA) of RNA-seq data. Cluster dendrograms are obtained from VST-transformed data. An euclidean distance is computed between samples and the dendrograms are built upon the Ward criterion. (a) RNA-seq data from half-brains of 17 month-old Bahd1-WT (WT1c, WT2c, WT3c) vs. Bahd1-KO (KO33c, KO56c, KO108c) mice. (b) RNA-seq data from whole brain of E16.5 embryos of Bahd1-WT (WT105c, WT112c, WT123c) vs. Bahd1-KO (KO71c, KO74c, KO78c) mice. (c) Analysis of DEGs between Bahd1-KO vs. Bahd1-WT embryonic brains, as in Fig 1A but without the KO78c outlier. (DOCX) [file pone.0232789.s003.docx]

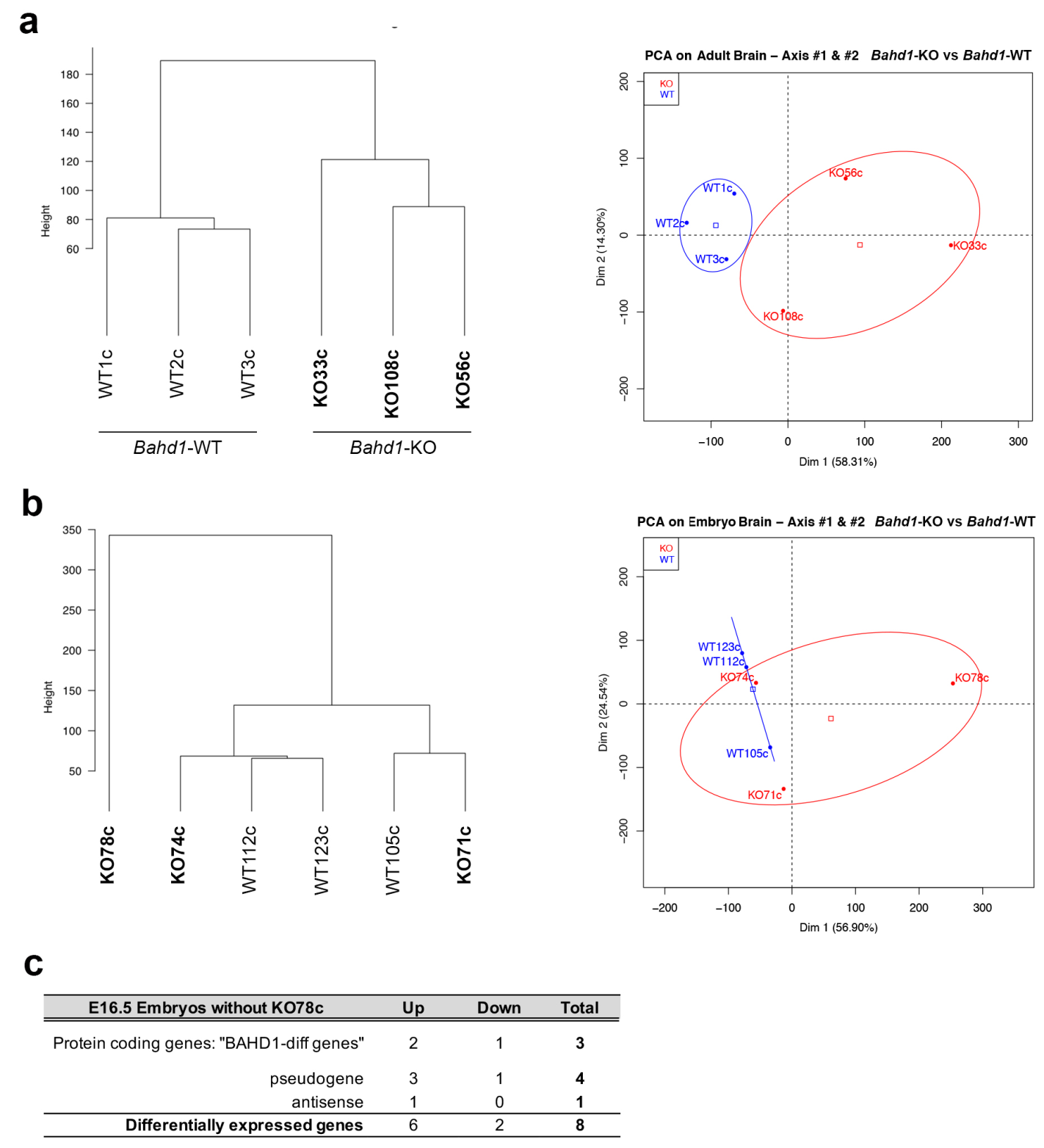


**S2 Fig. Transcriptome analysis of *Bahd1*^-/-^ brains (KO) in comparison with *Bahd1*^+/+^ (WT) brains.** (**a, b**) Euclidian hierarchical clustering and Principal Component Analysis (PCA) of RNA-seq data. Cluster dendrograms are obtained from VST-transformed data. An euclidean distance is computed between samples and the dendrograms are built upon the Ward criterion. (**a**) RNA-seq data from half-brains of 17 month-old *Bahd1*-WT (WT1c, WT2c, WT3c) *vs.* *Bahd1*-KO (KO33c, KO56c, KO108c) mice. (**b**) RNA-seq data from whole brain of E16.5 embryos of *Bahd1*-WT (WT105c, WT112c, WT123c) *vs.* *Bahd1*-KO (KO71c, KO74c, KO78c) mice. (**c**) Analysis of DEGs between *Bahd1*-KO *vs.* *Bahd1*-WT embryonic brains, as in Fig. 1a but without the KO78c outlier.
