# Supplementary material for: BAHD1 haploinsufficiency results in anxiety-like phenotypes in male mice
Source: PLoS One. 2020 May 14;15(5):e0232789. doi: 10.1371/journal.pone.0232789 (PMC7224496; doi:10.1371/journal.pone.0232789)
Supplement: S3 Fig — Data were extracted from Human Protein Atlas (HPA) available from v18.1.proteinatlas.org (www.proteinatlas.org). Specific image for GRIN, BAHD1 and RCOR1 and can be found at: https://www.proteinatlas.org/ENSG00000176884-GRIN1/tissue. https://www.proteinatlas.org/ENSG00000140320-BAHD1/tissue. https://www.proteinatlas.org/ENSG00000089902-RCOR1/tissue. 37 tissues have been analyzed by RNA-seq to estimate the transcript abundance of each protein-coding gene (for a total of 172 tissue samples) [11]. HPA RNA-seq tissue data is reported as mean TPM (protein-coding transcripts per million), corresponding to mean values of the different individual samples from each tissue. Color-coding is based on tissue groups. For tissue type, the average TPM value for replicate samples was used as abundance score. The threshold level to detect presence of a transcript for a particular gene was set to ≥ 1 TPM. GRIN1 belongs to “Tissue enriched” category of genes (expression in one tissue at least five-fold higher than all other tissues/cell lines) and is enriched in the brain. BAHD1 and RCOR1 belong to the “Expressed in all tissues” category of genes (≥ 1 TPM in all tissues/cell lines). (DOCX) [file pone.0232789.s004.docx]

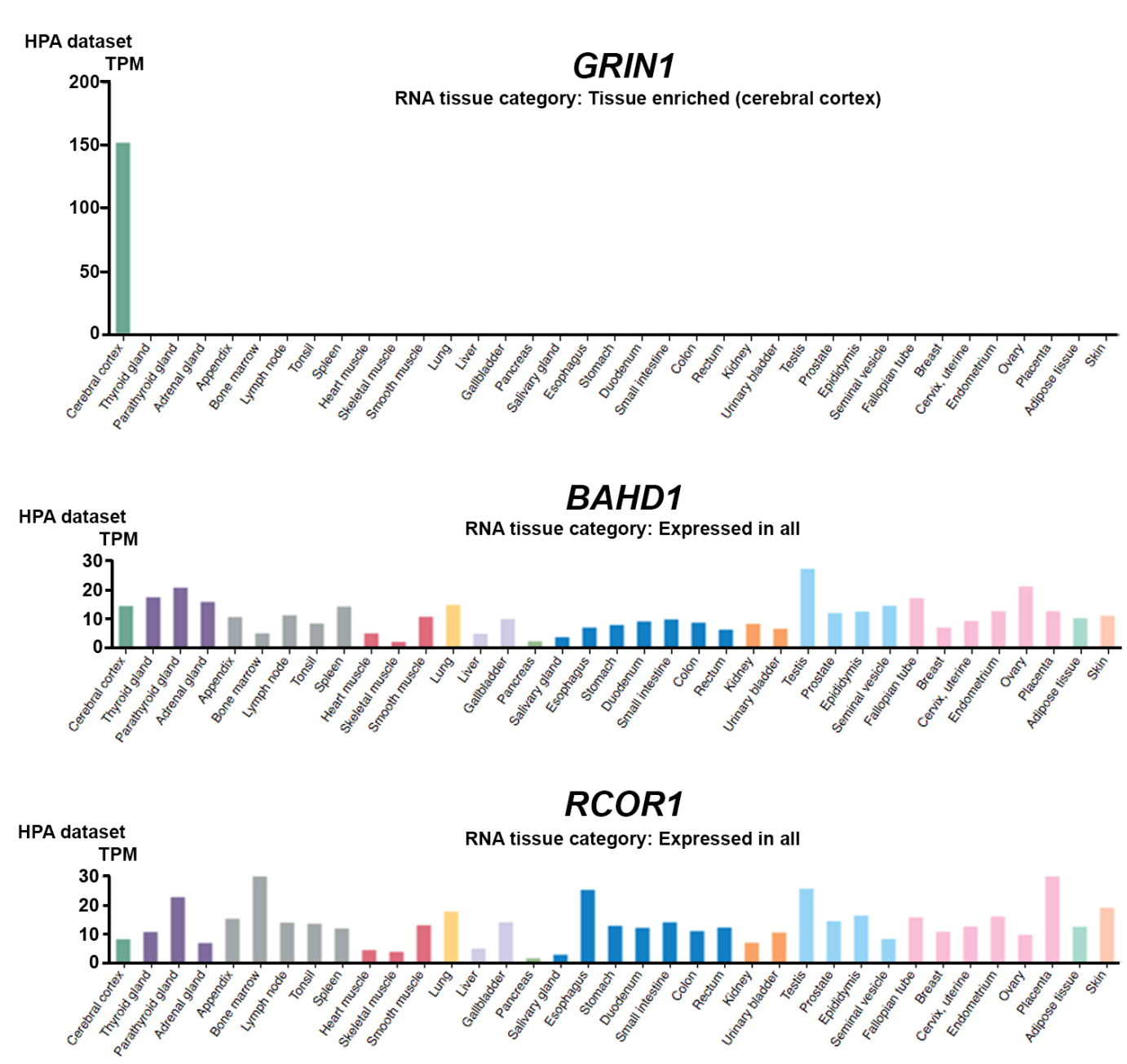


**S3 Fig. *BAHD1* gene expression levels in human tissues in comparison with *GRIN1* and *RCOR1.*** Data were extracted from Human Protein Atlas (HPA) available from v18.1.proteinatlas.org ([www.proteinatlas.org](https://www.proteinatlas.org/)). Specific image for *GRIN*, *BAHD1* and *RCOR1* and can be found at:

<https://www.proteinatlas.org/ENSG00000176884-GRIN1/tissue>

https://www.proteinatlas.org/ENSG00000140320-BAHD1/tissue

https://www.proteinatlas.org/ENSG00000089902-RCOR1/tissue

37 tissues have been analyzed by RNA-seq to estimate the transcript abundance of each protein-coding gene (for a total of 172 tissue samples) [11]. HPA RNA-seq tissue data is reported as mean TPM (protein-coding transcripts per million), corresponding to mean values of the different individual samples from each tissue. Color-coding is based on tissue groups. For tissue type, the average TPM value for replicate samples was used as abundance score. The threshold level to detect presence of a transcript for a particular gene was set to ≥ 1 TPM. *GRIN1* belongs to “Tissue enriched” category of genes (expression in one tissue at least five-fold higher than all other tissues/cell lines) and is enriched in the brain. *BAHD1* and *RCOR1* belong to the “Expressed in all tissues” category of genes (≥ 1 TPM in all tissues/cell lines).
