# Supplementary material for: BAHD1 haploinsufficiency results in anxiety-like phenotypes in male mice
Source: PLoS One. 2020 May 14;15(5):e0232789. doi: 10.1371/journal.pone.0232789 (PMC7224496; doi:10.1371/journal.pone.0232789)
Supplement: S4 Fig — RNA profiling data sets were generated by the Mouse ENCODE project [12] from C57BL/6 mice tissues of two biological replicates. Graphs are adapted from those released in the NCBI database (https://www.ncbi.nlm.nih.gov) for Grin1 (Gene ID 14810), Bahd1 (Gene ID: 228536) and Rcor1 (Gene ID: 217864). Histograms represent RNA-seq results reported as RPKM (Reads Per Kilobase Million). Adult tissues were taken from 8-week old littermates. Embryonic tissues of the Central nervous system (CNS) were taken from stage E11.5, E14 and E18 littermates. (DOCX) [file pone.0232789.s005.docx]

**
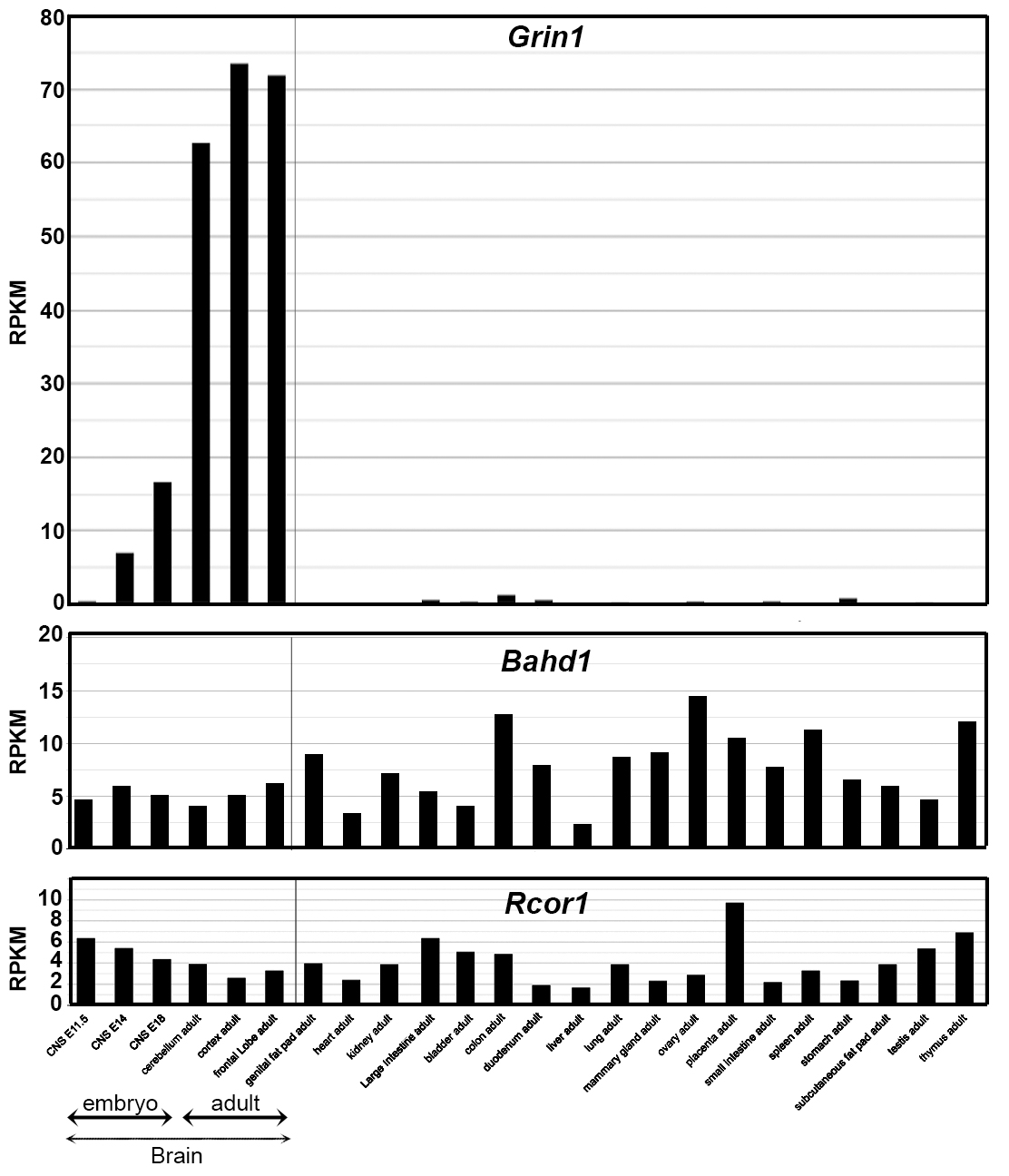
**

**S4 Fig. Expression profiling of *Grin1*, *Bahd1* and *Rcor1* in mouse tissues.** RNA profiling data sets were generated by the Mouse ENCODE project [12] from C57BL/6 mice tissues of two biological replicates. Graphs are adapted from those released in the NCBI database (<https://www.ncbi.nlm.nih.gov>) for *Grin1* (Gene ID 14810), *Bahd1* (Gene ID: 228536) and *Rcor1* (Gene ID: 217864). Histograms represent RNA-seq results reported as RPKM (Reads Per Kilobase Million). Adult tissues were taken from 8-week old littermates. Embryonic tissues of the Central nervous system (CNS) were taken from stage E11.5, E14 and E18 littermates.
