# Supplementary material for: BAHD1 haploinsufficiency results in anxiety-like phenotypes in male mice
Source: PLoS One. 2020 May 14;15(5):e0232789. doi: 10.1371/journal.pone.0232789 (PMC7224496; doi:10.1371/journal.pone.0232789)
Supplement: S5 Fig — Masks derived from the ISH results (Left), and the corresponding atlas section (Right) are shown. The regions with the highest BAHD1 expression are pointed with arrows (Hippocampal Formation (HPF), Olfactory areas (OLF) and Isocortex). (Adapted from https://mouse.brain-map.org) [13]. (DOCX) [file pone.0232789.s006.docx]

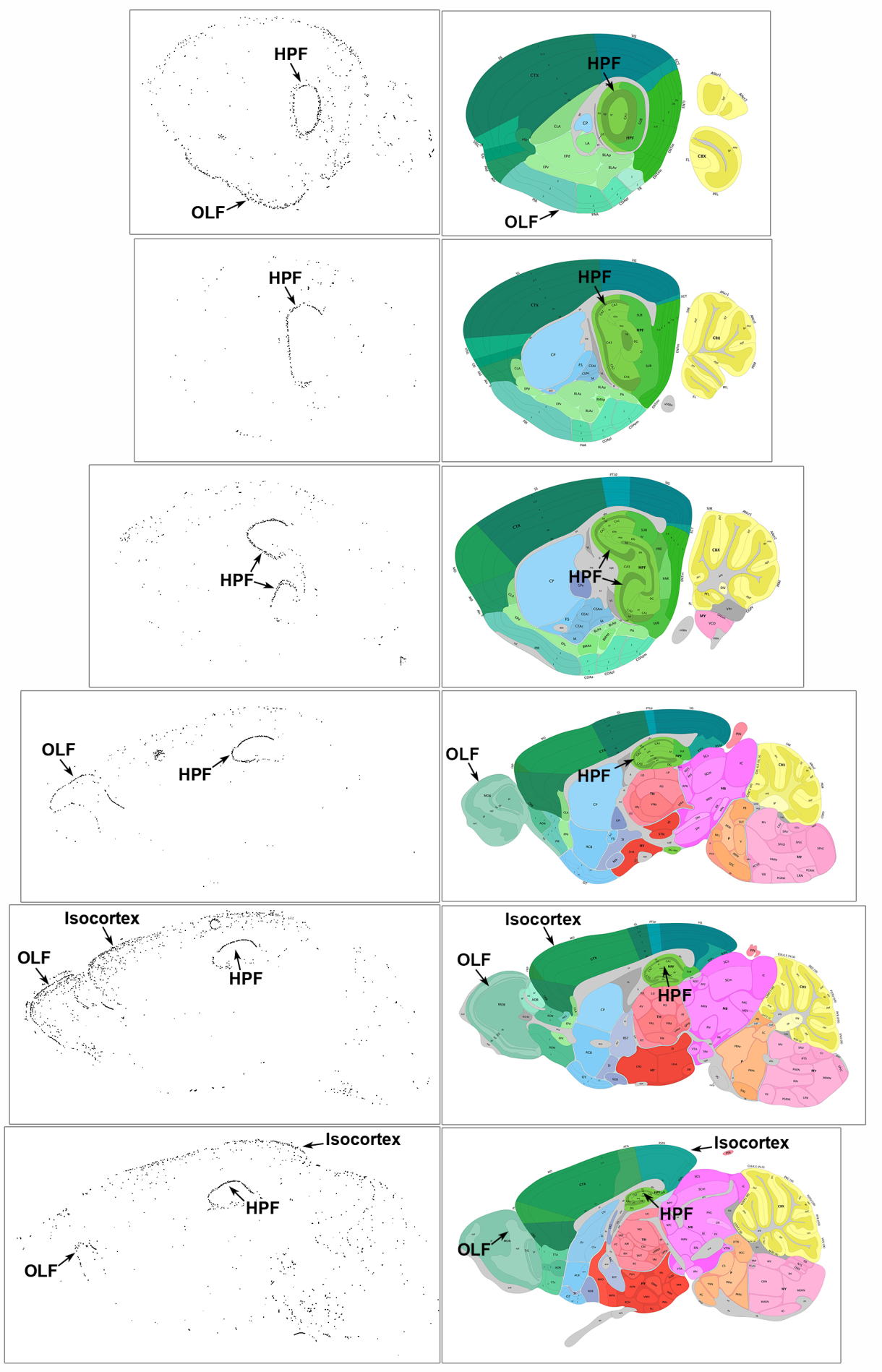


**S5 Fig. *BAHD1* expression in a set of sagittal sections from the Allen Mouse Brain Atlas**. Masks derived from the ISH results (*Left*), and the corresponding atlas section (*Right*) are shown. The regions with the highest *BAHD1* expression are pointed with arrows *(*Hippocampal Formation (HPF), Olfactory areas (OLF) and Isocortex).

(Adapted from https://mouse.brain-map.org)[13]
