# Supplementary material for: BAHD1 haploinsufficiency results in anxiety-like phenotypes in male mice
Source: PLoS One. 2020 May 14;15(5):e0232789. doi: 10.1371/journal.pone.0232789 (PMC7224496; doi:10.1371/journal.pone.0232789)
Supplement: S6 Fig — Histograms show the relative Bahd1 expression in 7 WT and 7 HET mice (relative to WT1) (Left), and mean ± S.E.M of Bahd1 expression in all HET relative to all WT mice (Right) (**** p<0 .0001). (DOCX) [file pone.0232789.s007.docx]

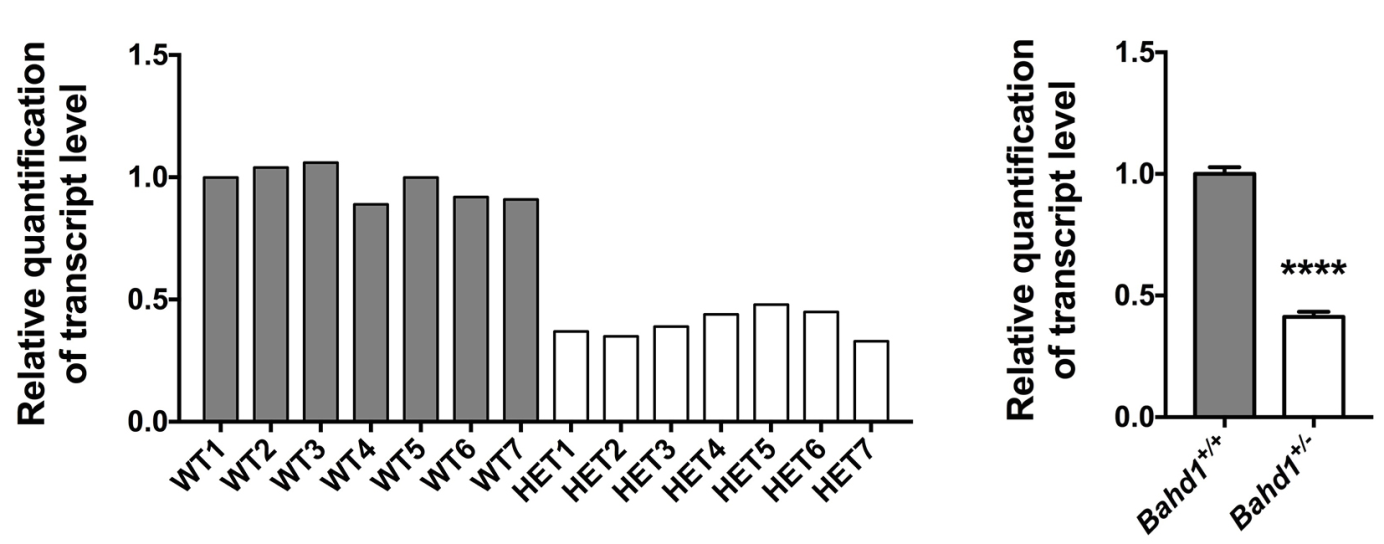


**S6 Fig.** **Relative expression levels of *Bahd1* mRNA in *Bahd1*-HET1 in comparison with *Bahd1*-WT brains by RT-qPCR**. Histograms show the relative *Bahd1* expression in 7 WT and 7 HET mice (relative to WT1) (*Left*), and mean ± S.E.M of *Bahd1* expression in all HET relative to all WT mice (*Right*) (**** *p*<0 .0001).
