# Supplementary material for: BAHD1 haploinsufficiency results in anxiety-like phenotypes in male mice
Source: PLoS One. 2020 May 14;15(5):e0232789. doi: 10.1371/journal.pone.0232789 (PMC7224496; doi:10.1371/journal.pone.0232789)
Supplement: S7 Fig — The auditory brainstem response test determines hearing sensitivity using evoked potential recordings in anaesthetized mice. ABR thresholds (decibels of sound pressure level, dB SPL) were recorded to the following frequencies and intensities of stimuli; 6kHz (20-85dB SPL), 12kHz (0-70dB SPL), 18kHz (0-70dB SPL), 24kHz (10-70dB SPL) and 30kHz (20-85dB SPL), presented in 5dB intervals. The Bahd1-Het2 mice show comparable ABR when compared to WT mice. (DOCX) [file pone.0232789.s008.docx]

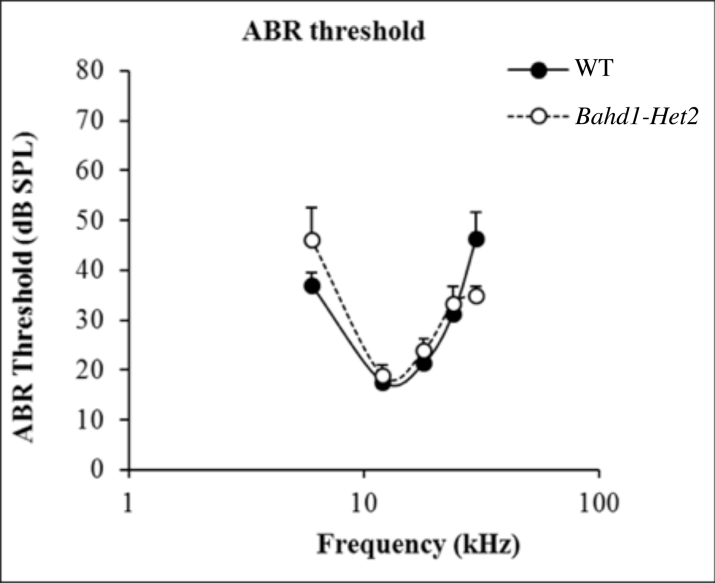


**S7 Fig. Audiograms of *Bahd1*-WT and *Bahd1*-Het2 mice.** The auditory brainstem response test determines hearing sensitivity using evoked potential recordings in anaesthetized mice. ABR thresholds (decibels of sound pressure level, dB SPL) were recorded to the following frequencies and intensities of stimuli; 6kHz (20-85dB SPL), 12kHz (0-70dB SPL), 18kHz (0-70dB SPL), 24kHz (10-70dB SPL) and 30kHz (20-85dB SPL), presented in 5dB intervals. The *Bahd1*-Het2 mice show comparable ABR when compared to WT mice.
